# Simple and efficient isolation of plant genomic DNA using magnetic ionic liquids

**DOI:** 10.1186/s13007-022-00860-8

**Published:** 2022-03-24

**Authors:** Miranda N. Emaus, Cecilia Cagliero, Morgan R. Gostel, Gabriel Johnson, Jared L. Anderson

**Affiliations:** 1 Department of Chemistry, Iowa State University, Ames, IA 50011 USA; 2 Dipartimento Di Scienza E Tecnologia del Farmaco, Università Degli Studi Di Torino, 10125 Turin, Italy; 3 Botanical Research Institute of Texas, Fort Worth, TX 76132 USA; 4 Smithsonian Institution, Suitland, MD 20746 USA; 5 Department of Chemistry, Iowa State University, 1605 Gilman Hall, Ames, IA 50011 USA

**Keywords:** Plant DNA isolation, One-step cell lysis, One-pot qPCR, Ionic liquids, *Arabidopsis thaliana* (L.) Heynh., *Quercus alba* L., *Aloe vera* L., *Nicotiana benthaminana* Domin

## Abstract

**Background:**

Plant DNA isolation and purification is a time-consuming and laborious process relative to epithelial and viral DNA sample preparation due to the cell wall. The lysis of plant cells to free intracellular DNA normally requires high temperatures, chemical surfactants, and mechanical separation of plant tissue prior to a DNA purification step. Traditional DNA purification methods also do not aid themselves towards fieldwork due to the numerous chemical and bulky equipment requirements.

**Results:**

In this study, intact plant tissue was coated by hydrophobic magnetic ionic liquids (MILs) and ionic liquids (ILs) and allowed to incubate under static conditions or dispersed in a suspension buffer to facilitate cell disruption and DNA extraction. The DNA-enriched MIL or IL was successfully integrated into the qPCR buffer without inhibiting the reaction. The two aforementioned advantages of ILs and MILs allow plant DNA sample preparation to occur in one minute or less without the aid of elevated temperatures or chemical surfactants that typically inhibit enzymatic amplification methods. MIL or IL-coated plant tissue could be successfully integrated into a qPCR assay without the need for custom enzymes or manual DNA isolation/purification steps that are required for conventional methods.

**Conclusions:**

The limited amount of equipment, chemicals, and time required to disrupt plant cells while simultaneously extracting DNA using MILs makes the described procedure ideal for fieldwork and lab work in low resource environments.

**Supplementary Information:**

The online version contains supplementary material available at 10.1186/s13007-022-00860-8.

## Background

The isolation of genomic DNA from plant tissue is an expensive, time-consuming, and delicate step underpinning downstream bioanalytical applications, such as barcoding for species identification [1], plant pathogen detection [2], and genetically modified organism (GMO) identification [3, 4]. Traditional plant cell lysis methods generally involve a cationic surfactant, such as cetrimonium bromide (CTAB) or sodium dodecyl sulfate (SDS) to solubilize the cell wall to release intracellular components, such as DNA, proteins, and lipids [5–7]. These lysis methods generally require significant heating (60–100 °C) as well as numerous centrifugation steps to isolate solid plant tissue. An additional purification step is then required to remove the lysis reagent. These detergents often inhibit nucleic acid amplification and detection, necessitating their removal [8]. Purification methods traditionally involve phenol–chloroform or spin column-based extractions, which generally utilize significant instrumentation and chemical reagents. To simplify plant DNA sample preparation, commercial kits have been developed, but these kits although effective still require access to laboratory equipment as well as lysis, binding, and wash buffers that often contain high concentrations of chaotropic salts and organic solvents. The combination of these aforementioned disadvantages results in lengthy sample preparation times, which is not ideal when high throughput analysis is required when operating in the field. Seeing how both cell lysis and DNA extraction are challenging to perform, a rapid and simple consolidation of these processes is highly desirable in the field of plant DNA analysis to improve sample throughput and reduce the amount of equipment required.

Ionic liquids (ILs) are molten salts with a melting temperature below 100 °C that exhibit several advantageous physico-chemical properties such as negligible vapor pressure, high thermal stability, high conductivity, and the ability for tunable properties through the judicious selection of the cation or anion component [9, 10]. Magnetic ionic liquids (MILs) are a subclass of ILs that contain a paramagnetic component in either the cation or anion structure [11–13]. While MILs exhibit similar characteristics to ILs, the paramagnetic component allows insoluble droplets to respond to an external magnetic field, permitting rapid collection of analyte-enriched MIL [14]. In contrast, ILs are often collected with a centrifuge [10]. The magnetic nature of MILs has facilitated the development of automated methods to perform extractions from multiple samples in a few minutes using a 96-well plate apparatus [15, 16]. MILs have been widely applied towards the extraction of nucleic acids [17–19]. In 2019, Marengo et al. first demonstrated the ability of Ni(II) and Co(II)-based MILs to extract genomic DNA from a plant cell lysate, significantly reducing the extraction time compared to conventional DNA extractions [20]. DNA purified by MILs has been shown to be sufficient for PCR amplification, sequencing, and DNA barcoding [17, 20, 21]. However, this study required a lengthy cell lysis procedure consisting of a heating step at 100 °C and a centrifugation step. Although this conventional SDS-based lysis method is effective for plant cell lysis, it is not ideal for field work in resource limited environments and still requires a substantial amount of time to implement. Recently, Emaus et al. reported a one-step cell lysis and DNA extraction protocol using MILs [22]. In this study, the hydrophobic MIL was simply dispersed in whole blood, and the DNA-enriched MIL was directly integrated into a PCR buffer where DNA was desorbed using the elevated temperatures required for thermocycling. The entire DNA sample preparation method required only 1 min compared to 40–60 min for the conventional spin column-based methods. The metal ion, ligand, and hydrophobic cation components of the MIL contributed towards the lysis of red and white blood cells. Applying this one-step sample preparation method to plant tissues would significantly improve sample throughput and allow for field analysis since instrumentation and additional chemicals are not required.

In this study, MILs and ILs were used to disrupt the plant cell wall and extract DNA in a single step. The trihexyl(tetradecyl)phosphonium ([P_6,6,6,14_^+^]) tris(hexafluoroacetylaceto)nickelate(II) ([Ni(hfacac)_3_^−^]) MIL, [P_6,6,6,14_^+^] tris(hexafluoroacetylaceto)colbaltate(II) ([Co(hfacac)_3_^−^]) MIL, and [P_6,6,6,14_^+^] bis[(trifluoromethyl)sulfonyl]imide ([NTf_2_^−^]) IL were used to develop three different approaches towards plant DNA sample preparation from *Arabidopsis thaliana* (L.) Heynh., *Quercus alba* L., *Aloe vera* L., and *Nicotiana benthaminana* Domin leaves. These three MIL-based methods (Table 1) constitute a simple, novel, and effective approach that requires significantly less time and equipment compared to conventional methods. The effectiveness of the solvents as chemical lysis reagents and DNA extraction media has the potential to drastically improve plant DNA analysis in the laboratory and field where it may be difficult to transport equipment.

**Table 1:** Abbreviated procedures for the three proposed lysis and DNA extraction methods discussed in this study

**Static extraction method**
Pipette 6 µL of MIL or IL onto 40 mg of plant tissue Allow the coated plant tissue to incubate Collect the hydrophobic liquid using a pipette Add 0.3 µL of either the DNA-enriched MIL or IL to the qPCR buffer
**Dispersive extraction method**
Suspend 40 mg of cut-up plant tissue in a Tris-based buffer Add 6 µL MIL to the sample Disperse the sample using a vortex Recover the MIL with a magnet Pipette 0.3 µL MIL into the qPCR buffer
**One-pot PCR method**
Grind plant tissue to a powder Add 1 mg plant tissue, 6 µL of MIL or IL, and the qPCR buffer to a reaction tube

## Results

### Static cell disruption and DNA extraction by ILs and MILs

**Fig. 1:**
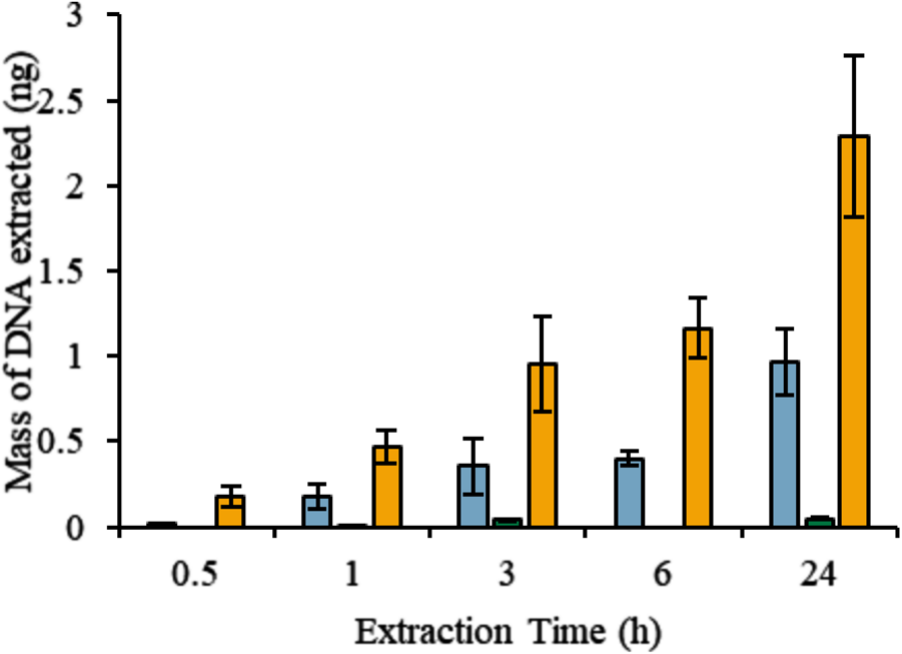
Mass of DNA recovered overtime after placing 6 µL of (blue) [P_6,6,6,14_^+^][Ni(hfacac)_3_^−^] MIL, (green) [P_6,6,6,14_^+^][Co(hfacac)_3_^−^] MIL, and (orange) [P_6,6,6,14_^+^][NTf_2_^−^] IL on 40 mg of *A. thaliana* tissue

**Fig. 2:**
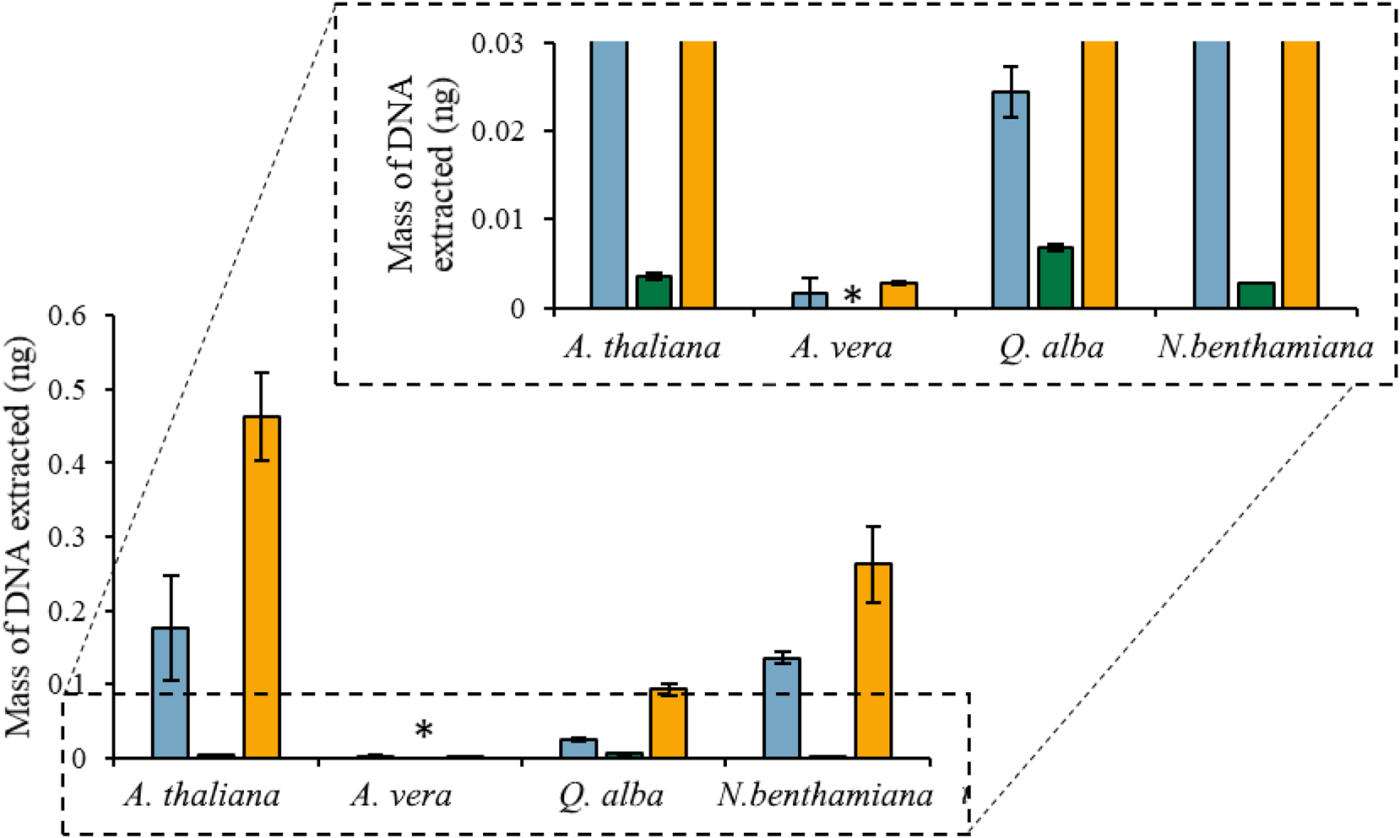
DNA extracted by placing 6 µL of (blue) [P_6,6,6,14_^+^][Ni(hfacac)_3_^−^], (green) [P_6,6,6,14_^+^][Co(hfacac)_3_^−^], and (orange) [P_6,6,6,14_^+^][NTf_2_^−^] on 40 mg of plant tissue for 1 h. *DNA recovered could not be quantified

Coating plant tissue with the [P_6,6,6,14_^+^][Ni(hfacac)_3_^−^] MIL, [P_6,6,6,14_^+^][Co(hfacac)_3_^−^] MIL, or [P_6,6,6,14_^+^][NTf_2_^−^] IL allows for cell disruption and DNA isolation with minimal pipetting steps. As shown in Fig. 1, a genomic DNA mass of 0.46 ± 0.10 ng was extracted with the [P_6,6,6,14_^+^][NTf_2_^−^] IL from 40 mg of intact *A. thaliana* tissue in 1 h. In comparison, an unquantifiable amount of DNA (Cq = 39.91 ± 0.09, signal was observed in 2/3 reactions by qPCR) was recovered when 6 µL of 2 mM Tris and 50 mM EDTA was placed on the leaf tissue for 1 h. This demonstrates that the hydrophobic solvent is disrupting the cell wall to release intracellular components as significantly more DNA is recovered with either the MIL or IL than Tris–EDTA buffer. The static plant cell lysis method was subsequently applied to *A. vera*, *Q. alba*, and *N. benthamiana* tissue, as shown in Fig. 2. This suggests that the hydrophobic MILs and IL can effectively disrupt various plant cells to release intracellular components despite the thick lignocellulosic cell wall that generally makes traditional plant cell lysis methods challenging.

The incubation time was examined to evaluate how much DNA could be recovered over time. Increasing the extraction time from 1 to 24 h resulted in higher amounts of DNA being recovered from all three MILs, as shown in Fig. 1. This suggests that although this method is simple and effective, it also requires a lengthy incubation time to isolate a maximum amount of DNA from intact plant tissue. In conventional plant cell lysis procedures, it is common to grind the plant material to increase the surface area and manually disrupt the cell wall prior to the addition of chemical lysis reagents [23]. Therefore, the effect of the size of the plant tissue utilized in the static extraction was investigated with 40 mg intact, cut-up (see Additional file 1: Figure S1 for size), and powdered plant tissue. Unfortunately, amplification was not observed when integrating 0.3 µL of MIL or IL exposed to powdered plant tissue into a qPCR buffer. As shown in Additional file 1: Figure S2, the colorless [P_6,6,6,14_^+^][NTf_2_^−^] IL turned green after being exposed to ground *A. thaliana* tissue for 30 min. The color change suggests that a large amount of impurities are being co-extracted by the [P_6,6,6,14_^+^][NTf_2_^−^] IL, likely causing PCR inhibition. As illustrated in Additional file 1: Figure S3, the largest amount of genomic DNA was recovered from 40 mg of cut-up *A. thaliana* tissue relative to intact tissue.

Drying plant tissue is a common approach to preserve nucleic acids during long term-storage and improve the amount of recovered nucleic acids [24, 25]. Therefore, a wide array of drying methods were investigated to evaluate their compatibility with the MIL-based method. As shown in Additional file 1: Figure S4, treating the plant tissue with isopropanol for 5 min or exposing the plant tissue to liquid nitrogen resulted in the highest amount of DNA recovered from the MIL (p > 0.05 by Student t-test). An additive effect in the amount of DNA isolated by the MIL was not observed when the plant tissue was exposed to isopropanol for 5 min followed by liquid nitrogen treatment. It is also important to note that the MIL-based cell disruption method was compatible with fresh plant tissue. This is momentous as it is difficult to transport large volumes of organic solvent or liquid nitrogen into the field. However, significantly less DNA was detected when sampling fresh plant tissue with the [P_6,6,6,14_^+^][Ni(hfacac)_3_^−^] MIL, [P_6,6,6,14_^+^][Co(hfacac)_3_^−^] MIL, and [P_6,6,6,14_^+^][NTf_2_^−^] IL compared to dry plant tissue (p > 0.05 by Student t-test). This shows that although fresh plant tissue is compatible with the MIL-based cell disruption and DNA extraction, drying the plant tissue improves the amount of DNA recovered. The effect of treating plant tissue with isopropanol for extended periods was also examined. As shown in Additional file 1: Figure S5, treating the plant tissue with isopropanol for 3 h resulted in the largest amount of recovered DNA; however, the amount of DNA drastically dropped after treating the plant tissue for periods of time longer than 3 h.

Conventional plant cell lysis methods utilize a heating step to improve the lysis efficiency and reduce the time required for lysis. Therefore, the effect of heating the plant tissue during the MIL-based cell disruption step was investigated, as shown in Additional file 1: Figure S6. Here, increasing the incubation temperature from 25 °C to 60 °C drastically improved the amount of DNA recovered from the [P_6,6,6,14_^+^][Ni(hfacac)_3_^−^] MIL and [P_6,6,6,14_^+^][NTf_2_^−^] IL. The amount of DNA recovered while heating the sample at 100 °C with the [P_6,6,6,14_^+^][Ni(hfacac)_3_^−^] MIL increased compared to the extraction at 60 °C. However, less DNA was recovered with the [P_6,6,6,14_^+^][NTf_2_^−^] IL at 100 °C compared to 60 °C which may be due to the co-extraction of PCR inhibitors when using the [P_6,6,6,14_^+^][NTf_2_^−^] IL.

### Dispersive cell disruption and DNA extraction using MILs

A dispersive cell disruption and DNA extraction method was developed to improve sample throughput by dramatically decreasing the extraction time. The [P_6,6,6,14_^+^][Ni(hfacac)_3_^−^] and [P_6,6,6,14_^+^][Co(hfacac)_3_^−^] MILs were chosen for the dispersive method since DNA-enriched MIL droplets can be rapidly recovered with a rod magnet. The *A. thaliana* leaves were cut up for all dispersive studies to ensure the highest amount of DNA is extracted while reducing the risk of co-extracting plant tissue. The concentration of EDTA (0–50 mM) in the suspension solution was first optimized; EDTA traditionally aids in cell lysis by chelating metal ions that stabilize the cell wall [23, 26]. As shown in Additional file 1: Figure S7, 50 mM EDTA was optimum with the [P_6,6,6,14_^+^][Ni(hfacac)_3_^−^] MIL, while the addition of EDTA to the buffer reduced the amount of DNA extracted by the [P_6,6,6,14_^+^][Co(hfacac)_3_^−^] MIL. The extraction time was subsequently optimized, as shown in Additional file 1: Figure S8. A 30 s vortex time was optimum in the dispersive method with the [P_6,6,6,14_^+^][Ni(hfacac)_3_^−^] MIL while 60 s was ideal for the [P_6,6,6,14_^+^][Co(hfacac)_3_^−^] MIL. A 6 µL aliquot of [P_6,6,6,14_^+^][Ni(hfacac)_3_^−^] and [P_6,6,6,14_^+^][Co(hfacac^−^] MILs was optimum for disrupting plant cells and capturing genomic DNA using the dispersive method, as shown in Additional file 1: Figure S9.

Although the described MIL-based methods were optimized with the DNA-enriched MIL in the qPCR assay, some bioanalytical detection methods, such as sequencing or Qubit detection, are not currently compatible with thermal desorption directly into the assay buffer. Therefore, desorption conditions were optimized to ensure the highest amount of DNA was recovered from the MIL into a Tris-based buffer. The effect of adding a non-ionic surfactant was investigated to reduce DNA adsorption to the plastic wall during thermal desorption [27]. The addition of 0.05% Tween20 to the desorption solution improved DNA recovery with a 10 min desorption time at 90 °C, as shown in Additional file 1: Figure S10. Previous studies have suggested that a sodium chloride-based desorption solution improves the amount of DNA recovered from the MIL [28]. Here, it was found that the largest amount of DNA was recovered from the [P_6,6,6,14_^+^][Co(hfacac)_3_^−^] MIL with a 200 mM NaCl, 0.05% Tween20 desorption solution while the addition of NaCl to the desorption solution did not assist in recovery DNA from the [P_6,6,6,14_^+^][Ni(hfacac)_3_^−^] MIL, as shown in Additional file 1: Figure S11. The desorption time was optimum at 10 min for both MILs (see Additional file 1: Figure S12).

**Fig. 3:**
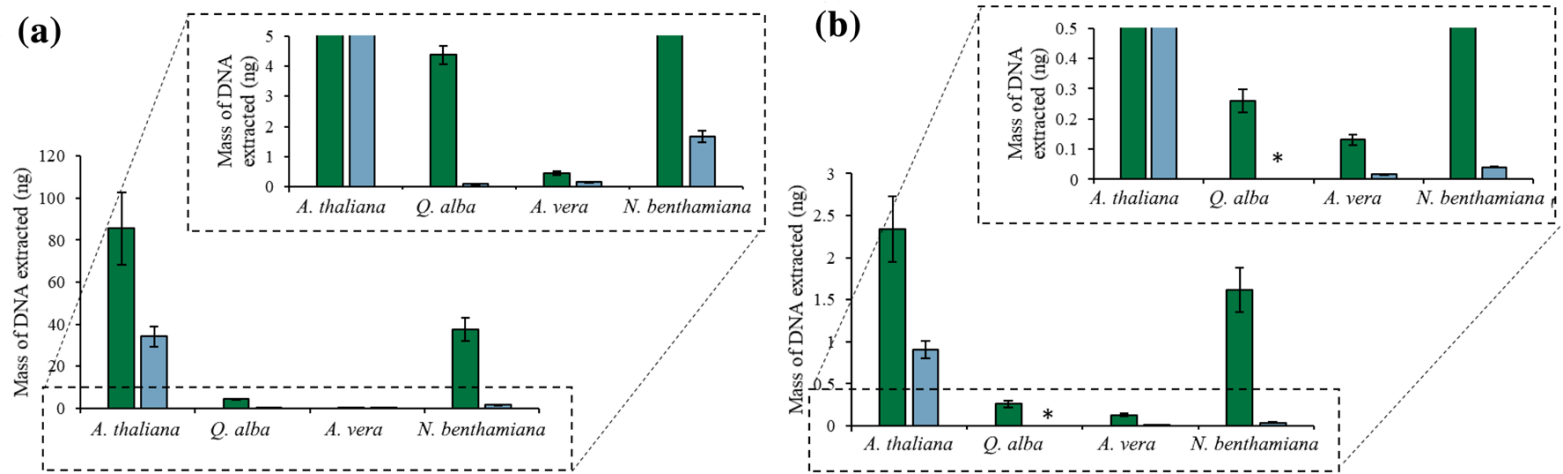
Amount of DNA recovered from the (green) [P_6,6,6,14_^+^][Ni(hfacac)_3_^−^] and (blue) [P_6,6,6,14_^+^][Co(hfacac)_3_^−^] MIL using the optimized dispersive lysis and DNA extraction method. Buffer volume: 0.5 mL; buffer composition with the [P_6,6,6,14_^+^][Ni(hfacac)_3_^−^] MIL: 2 mM Tris, 50 mM EDTA; buffer composition with the [P_6,6,6,14_^+^][Co(hfacac)_3_^−^] MIL: 2 mM Tris; mass of plant tissue: 40 mg; volume of MIL dispersed: 6 µL; dispersion time for the [P_6,6,6,14_^+^][Ni(hfacac)_3_^−^] MIL: 30 s; dispersion time for the [P_6,6,6,14_^+^][Co(hfacac)_3_^−^] MIL: 60 s. *DNA recovered could not be quantified

The dynamic one-step cell lysis and DNA extraction method was subsequently utilized on different plant tissues to demonstrate the versatility of the MIL-based method on different plant tissues. As shown in Fig. 3, the MIL-based method could extract large amounts of genomic DNA from *A. thaliana*, *N. benthamiana*, *Q. alba*, and *A. vera* tissue. Succulent tissue, in particular, is challenging to isolate DNA from due to the amount of polysaccharides forming insoluble complexes with nucleic acids [29] as well as the secondary metabolites of *Q. alba*. Although the least amount of DNA was recovered from *A. vera* compared to other plants with 0.44 ± 0.06 ng and 0.16 ± 0.01 ng of *A. vera* genomic DNA isolated by the [P_6,6,6,14_^+^][Ni(hfacac)_3_^−^] and [P_6,6,6,14_^+^][Co(hfacac)_3_^−^] MILs, respectively, amplification was successful, suggesting that the MIL-based lysis method can lyse a wide variety of plant tissues.

**Fig. 4:**
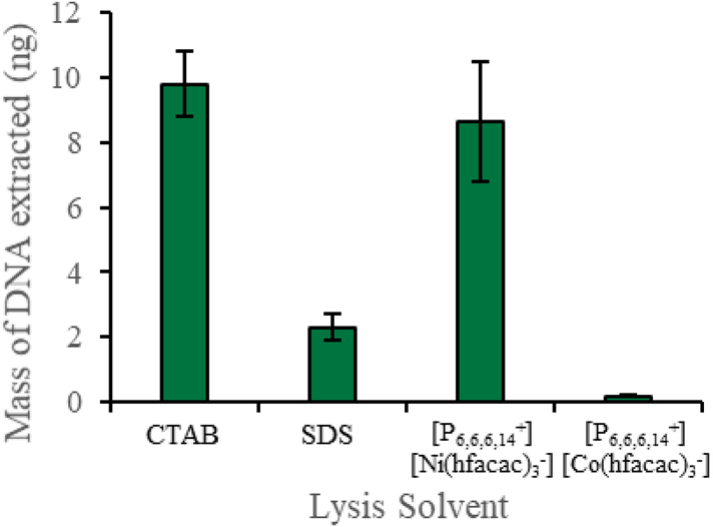
The amount of DNA recovered from a plant cell lysate generated using CTAB, SDS, [P_6,6,6,14_^+^][Ni(hfacac)_3_^−^] MIL, and [P_6,6,6,14_^+^][Co(hfacac)_3_^−^] MIL. Buffer volume: 0.5 mL; buffer composition with the [P_6,6,6,14_^+^][Ni(hfacac)_3_^−^] MIL: 2 mM Tris, 50 mM EDTA; buffer composition with the [P_6,6,6,14_^+^][Co(hfacac)_3_^−^] MIL: 2 mM Tris; mass of plant tissue: 40 mg; volume of MIL dispersed: 6 µL; dispersion time for the [P_6,6,6,14_^+^][Ni(hfacac)_3_^−^] MIL: 30 s; dispersion time for the [P_6,6,6,14_^+^][Co(hfacac)_3_^−^] MIL: 60 s

The amount of DNA released from plant cells by the [P_6,6,6,14_^+^][Ni(hfacac)_3_^−^] and [P_6,6,6,14_^+^][Co(hfacac)_3_^−^] MILs was compared to traditional lysis methods. In this experiment, the optimized dispersive lysis method was performed. The suspension buffer was recovered after the dispersion, and DNA was purified via isopropanol precipitation and quantified by qPCR. The amount of DNA recovered from the lysate generated with the [P_6,6,6,14_^+^][Ni(hfacac)_3_^−^] MIL was higher than the SDS lysis method and within error of the CTAB lysis procedure, as shown in Fig. 4. In addition, the MIL-based lysis and extraction required only 1 min without additional equipment to isolate nanogram amounts of DNA, and directly integrating the DNA-enriched MIL into the qPCR buffer significantly improves the recovery of DNA due to the low desorption volume.

The purity of the DNA recovered after the one-step cell lysis and DNA extraction was evaluated by determining the amplification efficiency of a BRAF DNA sequence spiked into the qPCR buffer. BRAF is a protooncogene that codes for the B-raf protein in humans and is not naturally found in plants, which is why it was used as a control sequence. A 6 µL aliquot of MIL was dispersed with 40 mg of *A. thaliana* plant tissue, and 0.3 µL of the recovered MIL was integrated into the qPCR assay. Ten-fold dilutions of a BRAF DNA sequence (98 bp) were added to the qPCR assay, and the amplification efficiency was determined from the standard curves using Eq. 1. The efficiency of the qPCR with the [P_6,6,6,14_^+^][Ni(hfacac)_3_^−^] and [P_6,6,6,14_^+^][Co(hfacac)_3_^−^] MIL previous used to lyse *A. thaliana* plant cells was 97.34% and 102.5%, respectively, as shown in Additional file 1: Figure S13. With qPCR, the amount of DNA should duplicate with each cycle, indicated by an amplification efficiency of 100%. Efficiencies ranging from 90–110% suggest that co-extracted components from the matrix do not inhibit the reaction.

1 $$ {\mkern 1mu} {\text{Efficiency}} = \left[ {10^{{\left( {{\raise0.7ex\hbox{${ - 1}$} \!\mathord{\left/ {\vphantom {{ - 1} {{\text{Slope}}}}}\right.\kern-\nulldelimiterspace} \!\lower0.7ex\hbox{${{\text{Slope}}}$}}} \right)}}  - 1} \right] \times {{100}}\%  $$

Quantification of genomic plant DNA recovered from the MIL was performed by qPCR and Qubit detection. The Qubit HS dsDNA assay is a fluorescence, non-specific DNA quantification method designed to be resilient to impurities that inhibit PCR detection, such as ethanol and chloroform [8]. As shown in Additional file 1: Figure S14, the amount of genomic *A. thaliana* DNA quantified by qPCR and the Qubit HS dsDNA assay were within error, suggesting that MILs do not inhibit Qubit detection even after dispersing the MIL in a plant cell suspension.

DNA integrity within the MIL over time was examined by performing agarose gel electrophoresis. Previous studies have suggested that plasmid DNA extracted from a DNase-rich environment is stable within a hydrophobic MIL solvent [30]. Salmon testes DNA (stDNA) (20 pg·µL^−1^) was spiked into the sample solution containing 40 mg of cut-up *A. thaliana* plant tissue, and the genomic DNA was extracted using the [P_6,6,6,14_^+^][Ni(hfacac)_3_^−^] MIL. The DNA-enriched MIL was incubated at 25 °C prior to agarose gel electrophoresis detection. After 24 h, the stDNA recovered from the MILs could still be visualized via gel electrophoresis, as shown in Additional file 1: Figure S15. However, the band associated with stDNA was not visible after 1 week of incubation, suggesting that DNA extracted using the one-step plant cell lysis and DNA extraction can be briefly stored in the MIL prior to bioanalytical analysis.

### One-pot plant cell lysis and qPCR assay

To further improve sample throughput, plant tissue was directly integrated into the qPCR assay while using a MIL or IL to lyse cells and prevent PCR inhibition. Assay optimization had a 20 pg ITS amplicon (330 bp) spiked into the qPCR assay to ensure DNA would be present in the aqueous phase during reaction optimization. Initial attempts to integrate 1 mg of dried *A. thaliana* tissue into the qPCR buffer with 0.3 µL of [P_6,6,6,14_^+^][Ni(hfacac)_3_^−^] MIL resulted in the reaction turning brown during thermocycling and amplification was not detected. It was hypothesized that the reaction did not contain enough MIL to thoroughly coat the plant tissue, which allowed inhibitors to enter the aqueous qPCR buffer. Increasing the volume of MIL in the reaction (2–8 µL) resulted in a colorless reaction buffer after thermocycling, as shown in Additional file 1: Figure S16, and 6 µL was chosen as the optimal MIL volume as it was the lowest volume of MIL where the plant tissue was initially submerged in the hydrophobic solvent. To alleviate inhibition caused by adding 6 µL of [P_6,6,6,14_^+^][Ni(hfacac)_3_^−^] MIL into the qPCR buffer, an additional 5 mM MgCl_2_ and 2 × SYBR green I was required. The annealing temperature also had to be reduced from 65 °C to 60 °C, as the addition of the hydrophobic MIL to the assay decreases the annealing temperature of DNA [18]. However, the reaction with 6 µL of [P_6,6,6,14_^+^][Ni(hfacac)_3_^−^] still failed to amplify with 1 mg *A. thaliana* tissue and qPCR conditions were further optimized.

It was noted that the plant tissue migrated to the interface between the hydrophobic MIL and aqueous qPCR buffer during thermocycling, and the lysate contaminated the aqueous buffer turning the reaction buffer brown. Plant tissue can float due to air between the cell walls [31], and removing this air may help relieve inhibition in the one-pot lysis. The effect of integrating 1 mg of intact, cut-up, or powdered plant tissue was examined to investigate whether this could prevent the tissue from migrating to the aqueous buffer. As shown in Additional file 1: Figure S17, when the plant tissue was not ground to a powder, the tissue would migrate to the interface between the hydrophobic MIL and aqueous qPCR buffer. As a result, the cut-up and intact tissue turned the assay brown. However, amplification was not observed with ground plant tissue and 6 µL [P_6,6,6,14_^+^][Ni(hfacac)_3_^−^] MIL with real-time fluorescence or gel electrophoresis readout again suggesting that further assay optimization was required to relieve inhibition.

**Fig. 5:**
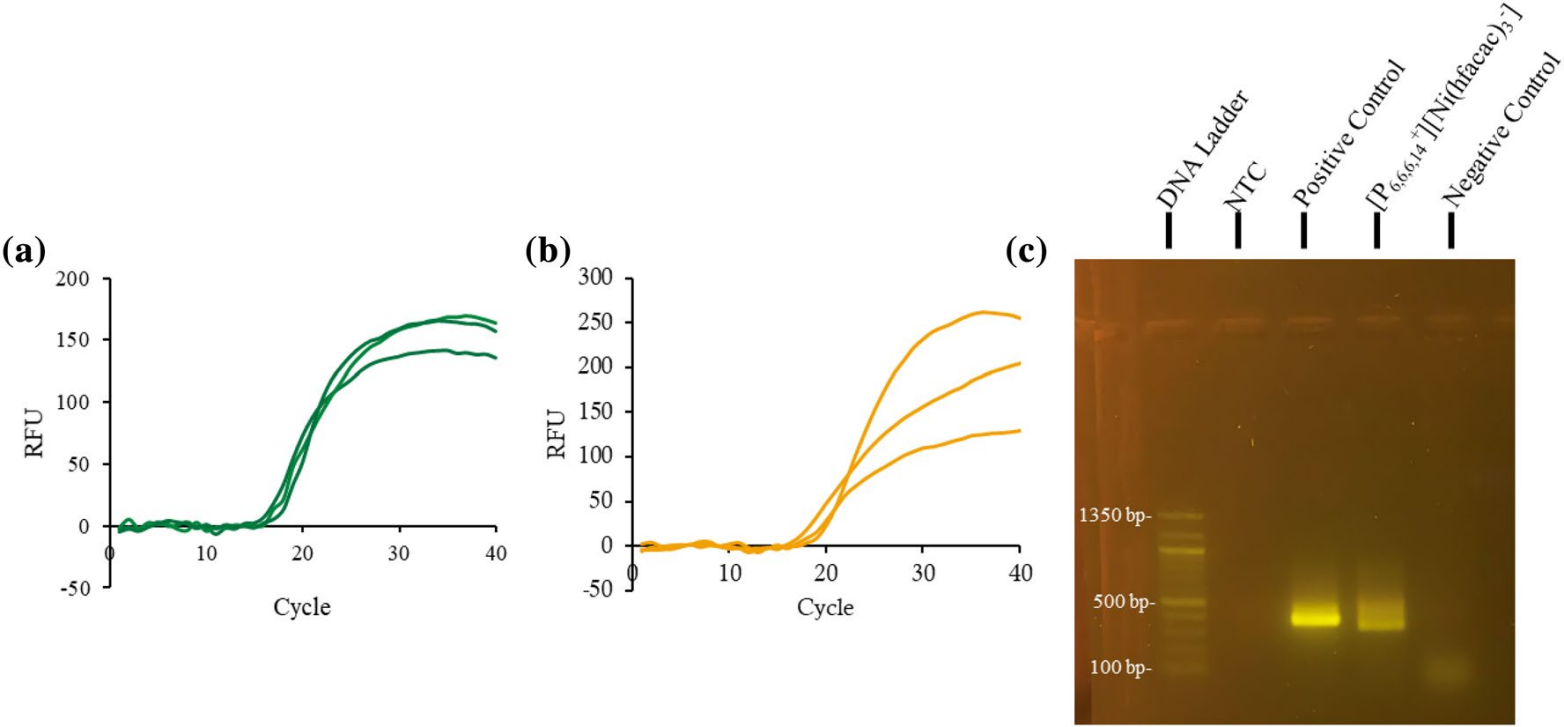
Amplification of 1 mg *A. thaliana* tissue and 6 µL [P_6,6,6,14_^+^][Ni(hfacac)_3_^−^] within the qPCR buffer **a** with 20 ng of ITS amplicon added to the assay and without the ITS spike with **b** qPCR and **c** gel agarose detection

Tween20 can relieve inhibition caused by polysaccharides from plant lysate [32], and introducing 0.05% Tween20 to the reaction buffer with 6 µL of [P_6,6,6,14_^+^][Ni(hfacac)_3_^−^] MIL and 1 mg powdered *A. thaliana* plant tissue recovered amplification. However, amplification frequently failed even with additional Tween20 (0.05–0.2%). As shown in Additional file 1: Figure S18, melt curves after the successful amplification of the ITS region indicated secondary structures were being formed during the reaction. DMSO can reduce the formation of secondary structures by disrupting hydrogen bond interactions [33]. Therefore, 2.5% DMSO (v/v) was added to the reaction buffer, which resulted in reliable amplification of the spiked ITS sequence (see Fig. 5a).

**Fig. 6:**
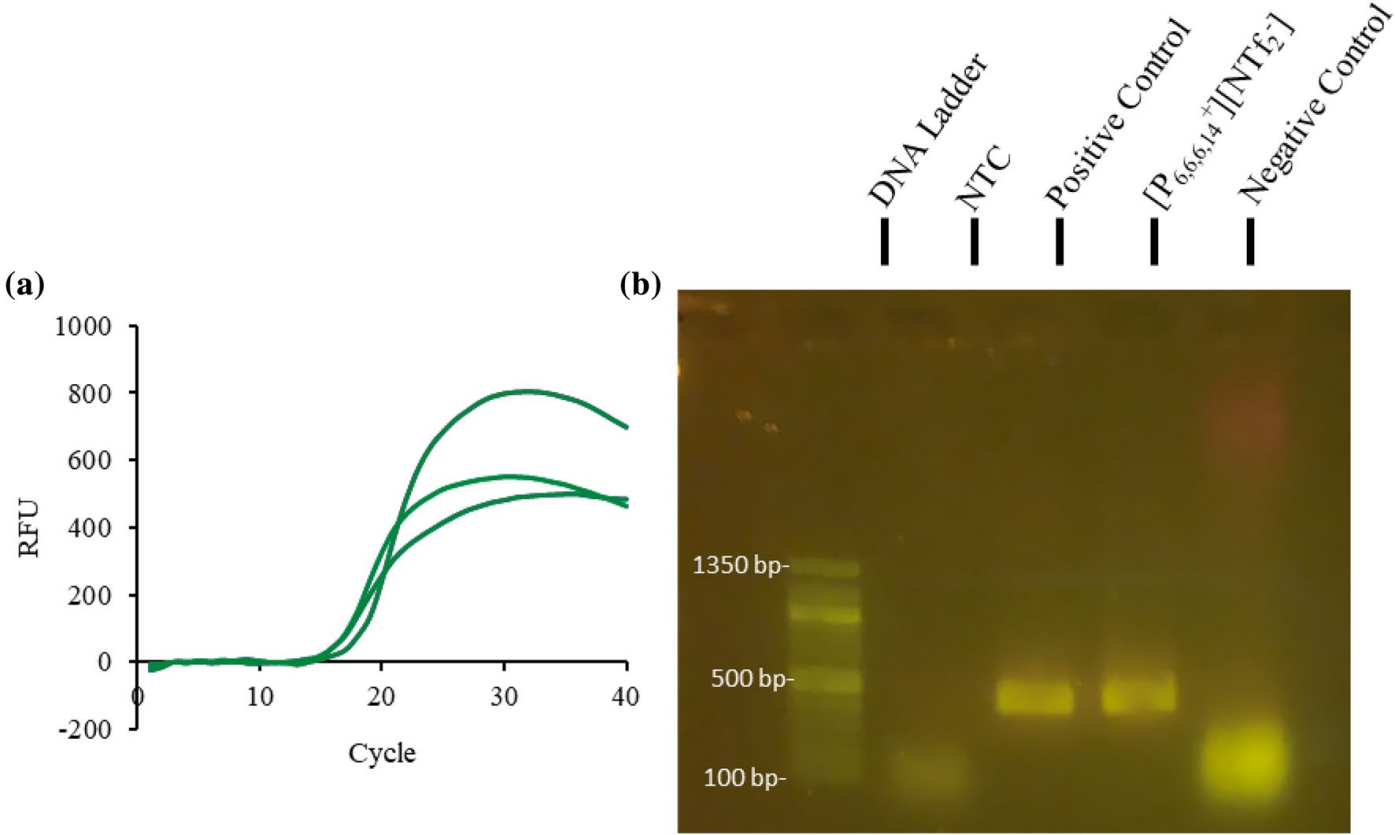
Fluorescence detection via **a** qPCR and **b** agarose gel electrophoresis with 1 mg of *A. thaliana* tissue and 6 µL of [P_6,6,6,14_^+^][NTf_2_^−^] IL integrated into the custom-qPCR assay

**Fig. 7:**
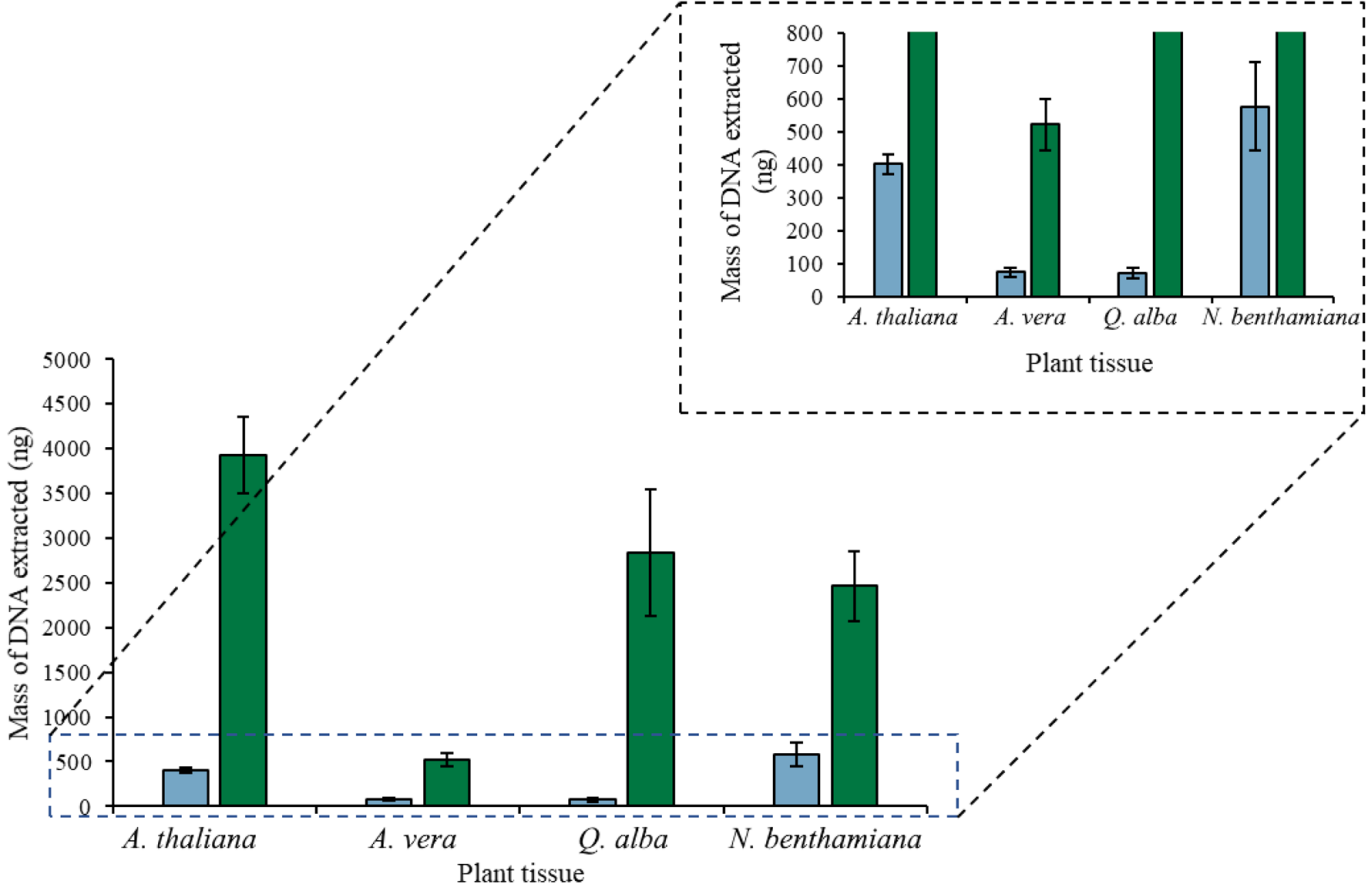
Integration of 0.5 mg of one of four plant tissues into a qPCR assay with 6 µL of (green) [P_6,6,6,14_^+^][Ni(hfacac)_3_^−^] MIL and (orange) [P_6,6,6,14_^+^][NTf_2_^−^] IL to facilitate cell lysis and prevent reaction inhibition

Removing the spiked ITS from the reaction still allowed for successful amplification, as shown in Fig. 5b, indicating that the MIL can disrupt the plant cells during thermocycling and still permit successful qPCR amplification and detection. The one-pot assay for the direct lysis and PCR amplification of genomic *A. thaliana* DNA was also optimized to contain 6 µL of [P_6,6,6,14_^+^][NTf_2_^−^] IL, as shown in Fig. 6. The reaction buffer for the one-pot qPCR assay with 6 µL of [P_6,6,6,14_^+^][NTf_2_^−^] IL and 1 mg powdered *A. thaliana* tissue required 1 × SSO Universal Supermix, 200 nM ITS primers, 2.5% DMSO, and 0.05% Tween20; the reaction was also run at 60 °C. Attempts to integrate larger masses of plant tissue into the qPCR assay were unsuccessful as the MIL did not sufficiently coat the plant tissue; nevertheless, 0.5 mg of *A. thaliana* plant tissue was successfully integrated into the qPCR assay with 6 µL of either [P_6,6,6,14_^+^][NTf_2_^−^] IL or [P_6,6,6,14_^+^][Ni(hfacac)_3_^−^] MIL, as shown in Fig. 7. The MIL-based method also worked with 1 mg of fresh *A. thaliana* tissue integrated into the qPCR assay, as shown in Additional file 1: Figure S19. This again demonstrates the potential of MILs and ILs for fieldwork as only a thermocycler is required.

**Fig. 8:**
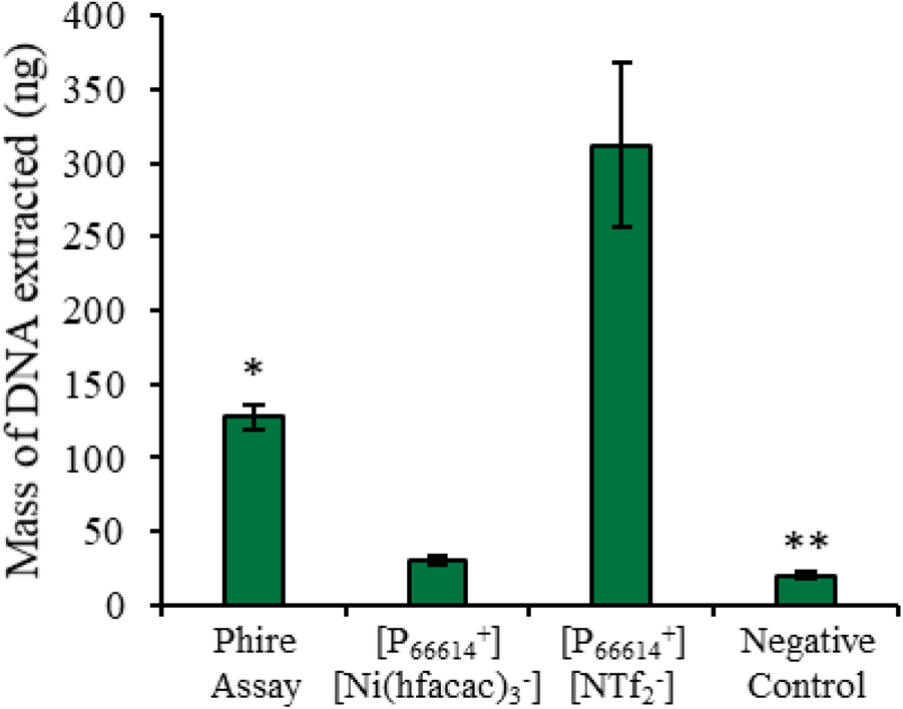
Comparison of the amount of DNA lysed during the different one-pot PCR assays. *Assay is not qPCR compatible. **Initial qPCR assay was unsuccessful

The amount of DNA released by the direct one-pot PCR assay was quantified and compared to reactions that contained 1 mg powdered *A. thaliana* tissue without the MIL or IL. Without the hydrophobic solvent, amplification was not observed; therefore, the lysate was diluted 100-fold prior to qPCR amplification to quantify the amount of DNA released during the initial thermocycling method. The MIL-based one-pot assay was also compared to the Phire assay buffer (Fisher Scientific). The Phire assay is a commercial one-pot PCR assay that allows up to 1 mg of plant tissue in the reaction buffer due to a custom-designed DNA polymerase. However, the Phire assay is not qPCR compatible and requires an endpoint detection method, such as gel electrophoresis (see Additional file 1: Figure S220). Therefore, the Phire assay was performed without primers to prevent amplification, and a 1 µL aliquot of the lysate from the Phire assay was added to the SSO Universal Supermix for qPCR quantification. More DNA was detected with the [P_6,6,6,14_^+^][NTf_2_^−^] IL, [P_6,6,6,14_^+^][Ni(hfacac)_3_^−^] MIL, and Phire assay compared to simply placing the plant tissue into the qPCR buffer and using the temperature profile for PCR to disrupt the plant cells. The [P_6,6,6,14_^+^][NTf_2_^−^] IL was most successful at lysing the plant cells with 311.8 ± 55.9 ng of *A. thaliana* DNA detected, while only 127.7 ± 8.4 ng was detected after lysing 1 mg of plant tissue with the Phire assay, as shown in Fig. 8.

**Fig. 9:**
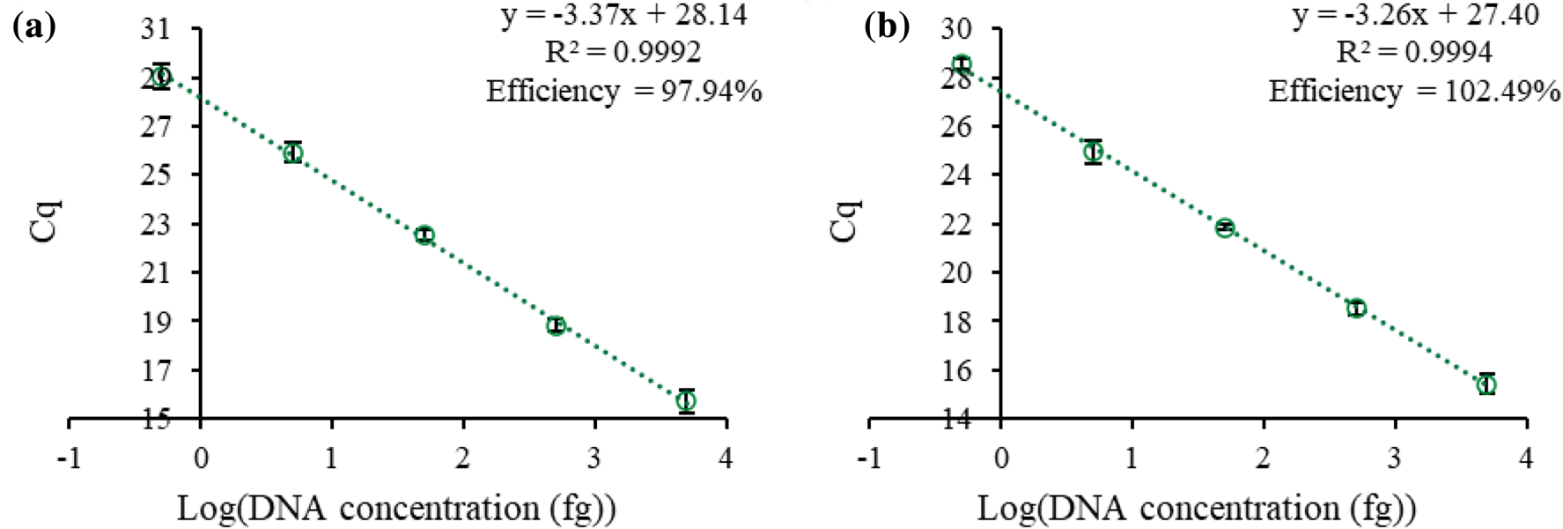
Standard curves generated with 6 µL of **a** [P_6,6,6,14_^+^][Ni(hfacac)_3_^−^] MIL and **b** [P_6,6,6,14_^+^][NTf_2_^−^] IL and 1 mg of *A. thaliana* plant tissue in the qPCR assay with a non-target DNA sequence spiked into the assay

The purity of the DNA in the aqueous phase was evaluated by spiking a BRAF DNA into the one-pot PCR assay containing 1 mg *A. thaliana* tissue to generate standard curves. As shown in Fig. 9, the efficiency associated with incorporating 6 µL of [P_6,6,6,14_^+^][NTf_2_^−^] IL and 1 mg plant tissue rose to 115.0%, suggesting some inhibition occurred due to the plant lysate. However, the efficiency associated with incorporating 6 µL of [P_6,6,6,14_^+^][Ni(hfacac)_3_^−^] MIL and 1 mg plant tissue was 97.05%, indicating that quantification is reliable with the one-pot qPCR assay with the [P_6,6,6,14_^+^][Ni(hfacac)_3_^−^] MIL.

Different plant tissues were integrated into the one-pot PCR reaction to disrupt the plant cells during the qPCR assay. The reaction was successful with the direct integration of 0.5 mg of *N. benthamiana*, *Q. alba*, and *A. vera* plant tissue, as shown in Fig. 9. Larger masses of plant tissue could not be successfully integrated into the direct qPCR assay as the MIL could no longer thoroughly coat the plant tissue due to larger volumes of plant tissue.

## Discussion

**Fig. 10:**
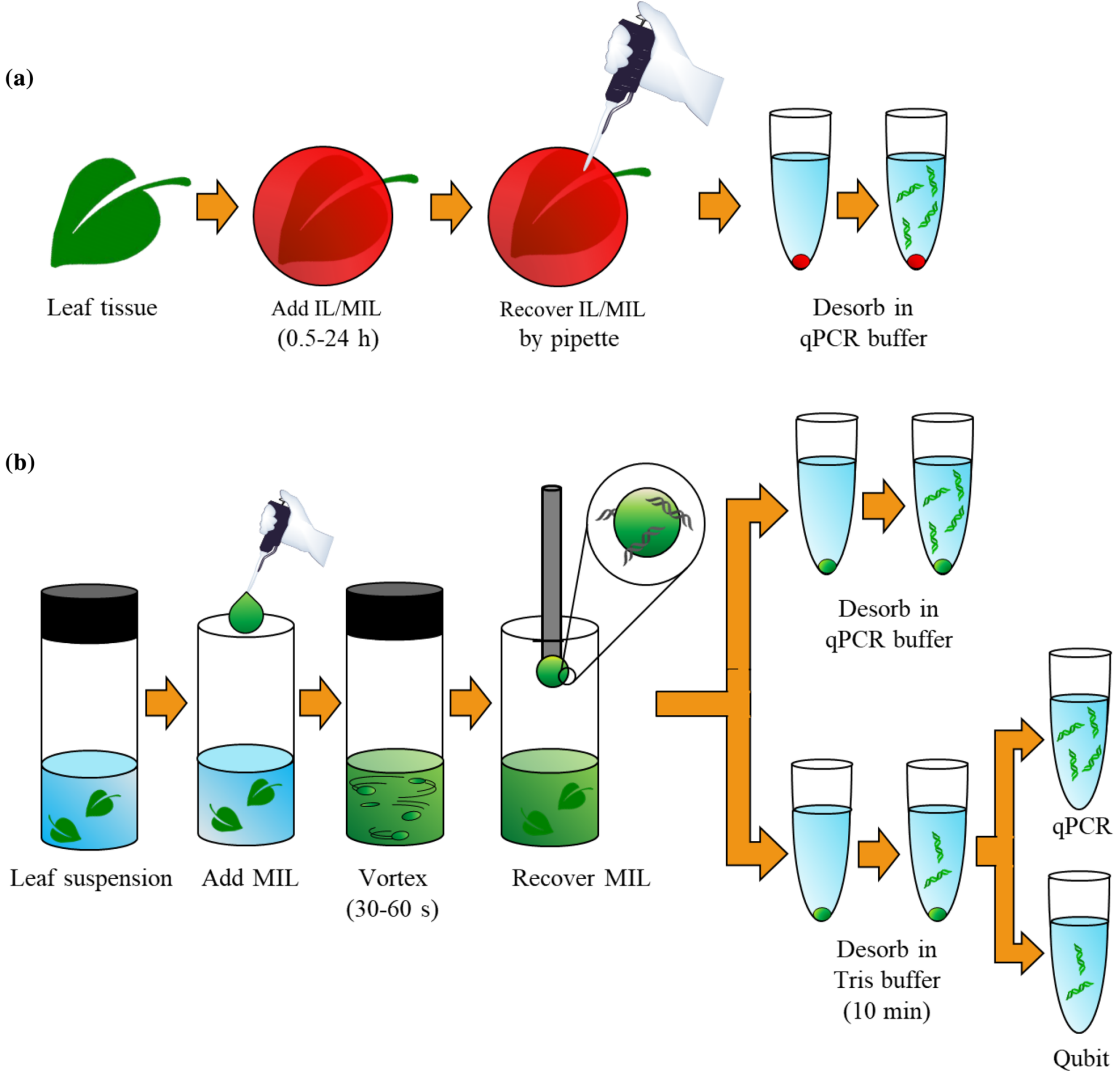
Schematic illustrating the **a** static and **b** dispersive one-step lysis and DNA extraction method using MILs

This study investigated hydrophobic ionic liquids (ILs) and magnetic ILs (MILs) (trihexyl(tetradecyl)phosphonium ([P_6,6,6,14_^+^]) tris(hexafluoroacetylaceto)nickelate(II) ([Ni(hfacac)_3_^−^]) MIL, [P_6,6,6,14_^+^] tris(hexafluoroacetylaceto)colbaltate(II) ([Co(hfacac)_3_^−^]) MIL, and [P_6,6,6,14_^+^] bis[(trifluoromethyl)sulfonyl]imide ([NTf_2_^−^], Additional file 1: Figure S20) as chemical plant cell lysis solvents. Three lysis and DNA extraction methods were developed and applied to *Arabidopsis thaliana* (L.) Heynh., *Quercus alba* L., *Aloe vera* L., and *Nicotiana benthaminana* Domin leaves (see Fig. 10 and Table 1). *A. thaliana* and *N. benthaminana* were selected as classical model plants while *Q. alba* and *A. vera* because of the challenges related to the DNA purification; indeed, *Q. alba* leaves are known for being rich in secondary metabolites that co-precipitate with DNA as well as the copious mucopolysaccarides that characterize *A. vera*. The three approaches include a static cell disruption method, a dispersive cell sample preparation method, and a one-pot qPCR assay.

In the static method, the hydrophobic solvent is pipetted directly onto plant tissue and allowed to incubate. A quantifiable amount of DNA can be detected in as little as 30 min in all the investigated plant materials with the [P_6,6,6,14_^+^][Ni(hfacac)_3_^−^] MIL, [P_6,6,6,14_^+^][Co(hfacac)_3_^−^] MIL, and [P_6,6,6,14_^+^][NTf_2_^−^] IL. The effect of the size of the plant tissue utilized in the static extraction was also investigated showing that the largest amount of genomic DNA can be recovered from fresh cut-up tissues relative to intact tissue. The developed MIL-based method does not require any equipment or require that the plant tissue be dry prior to sample preparation. The compatibility of the MIL-based cell disruption method with fresh plant tissue is of outmost importance for field applications as it is difficult to transport large volumes of organic solvent, liquid nitrogen or silica gel into the field. However, it should be remarked that less DNA was detected when sampling fresh plant tissue with the investigated IL and MILs compared to plant tissue dried with isopropanol and heated up to 60 °C showing that drying and applying heat to the sample aids in improving the amount of DNA recovered from the MIL.

The extraction time could be drastically reduced by dispersing the MIL in a suspension of plant tissue with only 0.5–1 min being required to lyse plant tissue and extract DNA. With the dispersive plant cell lysis method, the MIL can be dispersed in a suspension of plant tissue followed by desorption of DNA into Tris buffer at 90 °C or directly into the qPCR assay. The recovery of the nucleic acids from the MILs enables this approach to be compatible with downstream methods that are not compatible with thermal desorption directly into the assay buffer, such as sequencing or Qubit detection. On the other side, by integrating the MIL into the qPCR assay improved sample throughput by allowing the sample preparation step to occur in less than 1 min with comparable amounts of isolated DNA to conventional methods (SDS and CTAB lysis procedures). In contrast, conventional plant tissue lysis and DNA purification can require several hours since the procedure requires multiple centrifugation, filtering, and/or incubation steps as well as multiple reagents. Traditional methods also require bulky equipment, such as centrifuges and water baths, limiting their applicability in low-resource environments. The MIL-based cell disruption and DNA extraction methods were successfully applied to all the investigated plant tissues, including succulents. However, it is important to remark that DNA integrity experiments within the MIL over time showed that the DNA extracted using the one-step plant cell lysis and DNA extraction can be stored only for 24 h in the MIL prior to bioanalytical analysis. The compatibility of the proposed lysis protocol with quantitative analysis (even in presence of interfering DNA) was also investigated by qPCR and Qubit HS dsDNA assay showing good and comparable results suggesting that MILs do not inhibit fluorescence detection.

Finally, to further improve sample throughput and reduce the amount of required instrumentation, MIL-coated plant tissue was integrated directly into the PCR buffer. Indeed, DNA detection via nucleic acid amplification traditionally requires substantial purification to allow for reliable detection and quantification via PCR. However, circumventing DNA sample preparation could allow for high throughput analysis and field-based detection. *A. thaliana* leaves were therefore directly submerged by the hydrophobic solvent ([P_6,6,6,14_^+^][Ni(hfacac)_3_^−^] MIL) preventing inhibitors from entering with the aqueous reaction buffer while still lysing the plant tissue and therefore allowing the nucleic acids to be amplified by PCR. An amplification efficiency of 97.05% was obtained for *A. thaliana* tissue coated with 6 μL of [P_6,6,6,14_^+^][Ni(hfacac)3^−^] MIL and reacted with 1 × SSO Universal Supermix, 200 nM ITS primers, 2.5% DMSO, and 0.05% Tween20 at 60 °C but the sample amount should be limited to low plant mass (up to 0.5 or 1 mg) to allow IL/MILs to completely coat the tissue. The reaction is successful without specialized enzymes to combat inhibition caused by the plant lysate like the Phire assay, and unlike the Phire assay, fluorescence readout is compatible using the MILs or ILs to lyse the plant tissue allowing for real-time and quantitative detection. Again, the purity of the DNA obtained with the proposed one-pot approach as well as the applicability to different plant species (*A. thaliana, N. benthamiana*, *Q. alba*, and *A. vera*) was tested with successful results showing that quantification is reliable with the one-pot qPCR assay with the [P_6,6,6,14_^+^][Ni(hfacac)_3_^−^] MIL with a large set of samples.

## Conclusions

The one-step plant cell lysis and DNA extraction method and one-pot qPCR reaction using MILs described here provide a novel and simple alternative to rupturing difficult to lyse cells. The MIL and IL solvents alone can penetrate the plant tissue and extract enough DNA for Qubit and qPCR detection that outperforms commercial and conventional methods by reducing sample preparation time, allowing quantitative detection, and potentially leading to field analysis of plant tissue. The simple one-step nature of this sample preparation method is ideal for field and laboratory applications, and future studies should investigate the practicality of MIL-based plant cell lysis and DNA extraction in the field as well as the compatibility of the proposed approach with high throughput sequencing techniques such as next generation sequencing (NGS).

## Methods

### Chemicals and materials

Ammonium hydroxide (28–30% solution in water), CTAB (≤ 99%), glycerol (≤ 99%), 1,1,1,5,5,5-hexafluoroacetylacetone (99%), and nickel(II) chloride (98%) were purchased from Acros Organics (Morris Plains, NJ, USA). Anhydrous diethyl ether (99.0%) was purchased from Avantor Performance Materials Inc. (Center Valley, PA, USA). Modified plasmids (3.9 Kbp) containing a 210 bp insert (see Table S1) were obtained from Eurofin Genomics (Louisville, KY, USA). Trihexyl(tetradecyl)phosphonium chloride ([P_6,6,6,14_^+^][NTf_2_^−^]) (97.7%) was purchased from Strem Chemicals (Newburyport, MA, USA). Cobalt(II) chloride (97%), chloroform (> 99.8%), ethylenediaminetetraacetic acid (EDTA) (99.4–100.06%), isoamyl alcohol (≥ 98.0%), lithium bis[(trifluoromethyl)sulfonyl]imide ([Li^+^][NTf_2_^−^]), magnesium chloride hexahydrate (99.0–102.0%), methanol (99.7%), poly(vinylpolypryrrolidone) (~ 100 µm particle size), salmon testes DNA (stDNA) (20 Kbp), SDS (99%), and Tween20 were purchased from Sigma-Aldrich (St. Louis, MO, USA). SYBR Green I (10,000x) was purchased from Life Technologies (Carlsbad, CA, USA). Quick-load purple 100 bp and 1 Kbp DNA ladders were purchased from New England Biolabs (Ipswich, MA, USA). Primers (sequences shown in Table S1) were acquired from Integrated DNA Technologies (Coralville, IA, USA). Dimethyl sulfoxide (DMSO) (≥ 99.7%), sodium chloride (99.0%), optically clear PCR caps, tube strips, isopropanol (99.9%), and agarose were acquired from Thermo Fisher Scientific (Waltham, MA, USA). Tris–HCl was obtained from RPI (Mount Prospect, IL, USA). Neodymium magnets (0.66 T) were purchased from K&J Magnetics (Pipersville, PA, USA). Deionized water (18.2 MΩ cm), obtained from a Milli-Q water purification system, was used to prepare all aqueous solutions (Millipore, Bedford, MA, USA).

### Samples

Wild-type *Arabidopsis thaliana* (L.) Heynh seeds, obtained from VWR (Radnor, PA, USA), were grown at 25 °C under ambient light. Leaves were collected using sterilized scissors. An *Aloe vera* L. plant was purchased from Walmart (Sault Ste. Marie, MI, USA) and grown at 75 °C under ambient light. Leaves were collected using a sterilized knife, and the gel was removed prior to cell lysis. White oak (*Quercus alba* L.) leaves were collected in Hudsonville, MI. For these two plant samples a voucher specimen was deposited at the Iowa State University. *Nicotiana benthamiana* Domin leaves were obtained from the Department of Plant Pathology and Microbiology, Iowa State University, Ames, IA.

### MIL synthesis

Chemical structures of the three MILs are shown in Additional file 1: Figure S20. The [P_6,6,6,14_^+^][Ni(hfacac)_3_^−^] MIL, [P_6,6,6,14_^+^][Co(hfacac)_3_^−^] MIL, and [P_6,6,6,14_^+^][NTf_2_^−^] IL were synthesized and characterized using previously reported procedures [18, 34]. The solvents were stored at room temperature in a desiccator.

### qPCR assays and conditions

A Bio-Rad CFX96 Touch Real-time PCR (Hercules, CA, USA) was utilized for qPCR amplification of the internal transcribed spacer (ITS) region of plant DNA. The ITS region is conserved amongst plants allowing for the detection of genomic DNA from various plant tissues with a single set of primers [35]. Primer sequences were obtained from White et al. [35] Reactions containing 0.3–4 µL of [P_6,6,6,14_^+^][Ni(hfacac)_3_^−^] MIL, [P_6,6,6,14_^+^][Co(hfacac)_3_^−^] MIL, or [P_6,6,6,14_^+^][NTf_2_^−^] IL used the following amplification protocol: 10 min initial denaturation at 95 °C followed by 40 cycles comprised of a 15 s denaturation step at 95 °C and a 45 s annealing step at 65 °C. After each cycle, an optical detection step was used to track the reaction in real-time. qPCR reactions containing 6–8 µL of MIL or IL required an annealing temperature of 60 °C for successful amplification due to hydrophobic interactions between DNA and the MIL or IL destabilizing the DNA duplex [18]. Amplification of a BRAF DNA sequence with 0–6 µL of MIL or IL in the reaction had an initial 2 min hold at 95 °C prior to a 5 s denaturation step at 95 °C and a 30 s annealing step at 60 °C. The BRAF amplification protocol ran for 40 cycles and an optical detection step occurred after each cycle. The thermocycling parameters for the commercial Phire assay included an initial 5 min hold at 98 °C to lyse the plant tissue prior to a 5 s denaturation step at 98 °C and a 20 s annealing step at 62 °C. The Phire assay is not optimized for real-time detection so only end-point detection was used. After 40 cycles, the Phire assay included a hold at 4 °C to ensure that amplified DNA is stable prior to electrophoretic detection. All reaction parameters are summarized in Table S2. Melt curves of qPCR products were generated by heating the amplicons from 65 °C to 95 °C in 0.5 °C increments every 5 s.

The qPCR buffer for reactions that did not contain MIL or IL consisted of 1 × SSOAdvanced Universal SYBR Green Supermix and 200 nM of ITS primer for a total volume of 20 µL. Reactions containing 0.3 µL of [P_6,6,6,14_^+^][Ni(hfacac)_3_^−^] MIL, [P_6,6,6,14_^+^][Co(hfacac)_3_^−^] MIL, or [P_6,6,6,14_^+^][NTf_2_^−^] IL required 1 × SSOAdvanced Universal SYBR Green Supermix, 200 nM of ITS primers, and an additional 1 × SYBR green I for a total volume of 20 µL. Amplification of a non-target DNA sequence with 0.3 µL of [P_6,6,6,14_^+^][Ni(hfacac)_3_^−^] or [P_6,6,6,14_^+^][Co(hfacac)_3_^−^] MIL required 1 × SSOAdvanced Universal SYBR Green Supermix, 1 µM primers, and an additional 1 × SYBR green I. The one-pot PCR assay contained 1 mg of *A. thaliana* plant tissue with 6 µL of [P_6,6,6,14_^+^][Ni(hfacac)_3_^−^] MIL and required 1 × SSOAdvanced Universal SYBR Green Supermix, 200 nM of ITS primers, 0.05% Tween20, 2.5% DMSO, an additional 2 × SYBR green I, and an additional 5 mM MgCl_2_ for a total volume of 20 µL. Amplification with 1 mg of *A. thaliana* plant tissue and 6 µL of [P_6,6,6,14_^+^][NTf_2_^−^] IL required 1 × SSOAdvanced Universal SYBR Green Supermix, 200 nM of ITS primers, 0.05% Tween20, 2.5% DMSO, and an additional 2 × SYBR green I for a total volume of 20 µL. The commercial Phire assay consisted of a 50 µL reaction buffer of 1 × Phire Plant PCR buffer, 1 mg of *A. thaliana* tissue, 0.5 µM primers, and 0.4 µL of the Phire Hot Start II DNA Polymerase. Reaction volumes of 20 µL were not successful with the Phire assay. Amplification of the spiked BRAF DNA sequence with 6 µL of [P_6,6,6,14_^+^][Ni(hfacac)_3_^−^] MIL in the qPCR assay required 1 × SSOAdvanced Universal SYBR Green Supermix, 1 µM BRAF primers, 0.05% Tween20, an additional 2 × SYBR green I, and an additional 5 mM MgCl_2_. Integration of 6 µL of [P_6,6,6,14_^+^][NTf_2_^−^] IL into a qPCR assay to amplify a BRAF DNA sequence utilized a buffer consisting of 1 × SSOAdvanced Universal SYBR Green Supermix, 1 µM BRAF primers, 0.05% Tween20, and an additional 2 × SYBR green I. A summary of all custom-designed PCR assays is included in Table S3.

The quantitation cycle (Cq) was determined using the fluorescence threshold provided by Bio-Rad CFX Maestro software. Standard curves were constructed to determine the amount of genomic *A. thaliana*, *A. vera*, *Q. alba*, and *N. benthamiana* DNA recovered from the [P_6,6,6,14_^+^][Ni(hfacac)_3_^−^] MIL, [P_6,6,6,14_^+^][Co(hfacac)_3_^−^] MIL, and [P_6,6,6,14_^+^][NTf_2_^−^] IL by plotting the Cq against the log of the amount of DNA initially present in the reaction, as shown in Additional file 1: Figure S21.

### Qubit DNA quantification

A Qubit 2.0 fluorometer (ThermoFisher Scientific, Waltham, MA, USA) with the double-stranded DNA (dsDNA) high-sensitivity (HS) assay was used to quantify the amount of genomic DNA recovered from the MIL. Briefly, 5 µL of recovered *A. thaliana* DNA was added to 194 µL of the Qubit dsDNA HS buffer and 1 µL of the Qubit dsDNA HS reagent. After incubating at room temperature for 3 min to allow the fluorophore to bind to dsDNA, the genomic DNA was quantified using the fluorimeter.

### Agarose gel electrophoresis conditions

Agarose gel electrophoresis was performed using a Bethesda Research Laboratories H4 Horizontal Gel Electrophoresis system (Life Technologies) and a dual output power supply (Neo/Sci, Rochester, NY, USA) to examine the integrity of the ITS amplicon and genomic DNA extracted by the MIL. A 0.5% gel was used to visualize genomic DNA, and a 1% agarose gel was used to separate the ITS amplicon (330 bp) from PCR components due to the short size of the amplicon. All gels were run for 1.5 h at 70 V and were visualized using a Safe Imager 2.0 transilluminator (Invitrogen, Carlsbad, CA, USA).

### Plant cell lysis conditions using MILs and ILs

A static one-step cell lysis and DNA extraction approach is illustrated in Fig. 10a and was developed by placing 6 µL of [P_6,6,6,14_^+^][Ni(hfacac)_3_^−^] MIL, [P_6,6,6,14_^+^][Co(hfacac)_3_^−^] MIL, or [P_6,6,6,14_^+^][NTf_2_^−^] IL onto 40 mg of plant tissue. The MIL or IL coated leaf was incubated at 25 °C for 0.5–24 h. The DNA-enriched MIL was subsequently recovered, and 0.3 µL of MIL was added to the qPCR assay for DNA amplification and quantification. All plant tissue was dried at room temperature unless otherwise specified. All experiments were performed in triplicate.

The dispersive one-step lysis and DNA extraction method is illustrated in Fig. 10b. The extraction time, volume of MIL dispersed, and EDTA concentration of the suspension buffer was optimized to rapidly obtain pure DNA from the plant tissue. Cut plant tissue (40 mg) was added to a 5 mL glass vial and suspended in 0.5 mL of 2 mM Tris buffer. An optimized volume of MIL (i.e., 6 µL) was added to the leaf suspension and vortexed for a specific amount of time. After recovering the MIL with a rod magnet (0.7 T), the DNA-enriched MIL was washed once with water. DNA was desorbed from the MIL by integrating 0.3 µL of DNA-enriched MIL into the qPCR assay to allow desorption during thermocycling or by heating all of the recovered MIL at 90 °C for 10 min in an optimized desorption solution. All experiments were performed in triplicate with dry plant tissue.

Conventional plant cell lysis was performed using the CTAB lysis and extraction method reported by Doyle et al. [7] Briefly, 40 mg of dry plant tissue was ground to a powder and suspended in 0.5 mL of CTAB lysis buffer (2% (w/v) CTAB, 1.4 M NaCl, 20 mM EDTA, 100 mM Tris (pH 8.0)). After heating the tissue at 60 °C for 30 min, 0.5 mL of chloroform-isoamyl alcohol solution (24:1; v/v) was added to the lysate and manually mixed. The sample was centrifuged to ensure phase separation between the aqueous and organic phases. The DNA-enriched aqueous layer was recovered, and 1 mL isopropanol was added to the sample to precipitate DNA. The sample was centrifuged again for 10 min to pellet the genomic DNA. The pellet was dried and subsequently reconstituted in 2 mM Tris buffer for downstream bioanalytical detection. SDS lysis was performed using the procedure from Marengo et al. [20] In summary, 40 mg of ground plant tissue, 5 mg of polyvinylpolypyrrolidone (PVPP), 15 µL of 40 µg/mL RNase was exposed to 500 µL of 50 mM Tris–EDTA (pH 8) and 3 µM for 15 min at 100 °C. Afterwards, the sample was centrifuged at 13,000 rpm for 15 min, and the supernatant was collected for isopropanol purification.

## Supplementary Information

**Additional file 1.** Additional figures.

## Data Availability

The data sets supporting the results of this article are included within the article (Figs. 1, 2, 3, 4, 5, 6, 7, 8, 9 and 10) and in the supporting information.
