# Supplementary material for: Simple and efficient isolation of plant genomic DNA using magnetic ionic liquids
Source: Plant Methods. 2022 Mar 24;18:37. doi: 10.1186/s13007-022-00860-8 (PMC8943943; doi:10.1186/s13007-022-00860-8)
Supplement: Supplementary file 1 — Additional file 1. Additional figures. [file 13007_2022_860_MOESM1_ESM.docx]

**Electronic Supplemental Information**

**Simple and Efficient Isolation of Plant Genomic DNA using Magnetic Ionic Liquids**

Miranda N. Emaus, Cecilia Cagliero, Morgan R. Gostel, Gabriel Johnson, and
 Jared L. Anderson^*^

**Author details**

MNE: Department of Chemistry, Iowa State University, Ames, Iowa 50011, USA, email: [memaus@iastate.edu](mailto:memaus@iastate.edu)

CC: Dipartimento di Scienza e Tecnologia del Farmaco, Università degli Studi di Torino, I-10125, Turin, Italy, email: [cecilia.cagliero@unito.it](mailto:cecilia.cagliero@unito.it)

MRG: Botanical Research Institute of Texas, Fort Worth, Texas 76132, USA, email: [mgostel@brit.org](mailto:mgostel@brit.org)

GJ: Smithsonian Institution, Suitland, Maryland 20746, USA, email: [JohnsonG@si.edu](mailto:JohnsonG@si.edu)

JLA: Department of Chemistry, Iowa State University, Ames, Iowa 50011, USA, email: [andersoj@iastate.edu](mailto:andersoj@iastate.edu)

* Corresponding author:

Jared L. Anderson
Department of Chemistry

Iowa State University

1605 Gilman Hall
Ames, IA 50011
Tel.: +1 515-294-8356

E-mail address: andersoj@iastate.edu

**Table of Contents**

| Table S1 | p. S3 |
| --- | --- |
| Table S2 | p. S4 |
| Table S3 | p. S5 |
| Figure S1 | p. S6 |
| Figure S2 | p. S7 |
| Figure S3 | p. S8 |
| Figure S4 | p. S9 |
| Figure S5 | p. S10 |
| Figure S6 | p. S11 |
| Figure S7 | p. S12 |
| Figure S8 | p. S13 |
| Figure S9 | p. S14 |
| Figure S10 | p. S15 |
| Figure S11 | p. S16 |
| Figure S12 | p. S17 |
| Figure S13 | p. S18 |
| Figure S14 | p. S19 |
| Figure S15 | p. S20 |
| Figure S16 | p. S21 |
| Figure S17 | p. S22 |
| Figure S18 | p. S23 |
| Figure S19 | p. S24 |
| Figure S20 | p. S25 |
| Figure S21 | p. S26 |
| Figure S22 | p. S27 |

**Table S1:** Sequences of the ITS primers and DNA sequences used in this study.

| Name | Sequence |
| --- | --- |
| ITS 3 | 5’-GCA TCG ATG AAG AAC GCA GC-3’ |
| ITS 4 | 5’-TCC TCC GCT TAT TGA TAT GC-3’ |
| Forward Non-target Primer | 5’-TTCATGAAGACCTCACAGTAAA-3’ |
| Reverse Non-target Primer | 5’-GGATCCAGACAACTGTTCAA-3’ |
| 98 bp Non-target Sequence | 5’-TTCATGAAGACCTCACAGTAAAAATAGGTGAT TTTGGTCTAGCTACAGtGAAATCTCGATGGAGTGGG  TCCCATCAGTTTGAACAGTTGTCTGGATCC-3’ |
| 210 bp Insert | 5’-TATATTTCTTCATGAAGACCTCACAGTAAAAA TAGGTGATTTTGGTCTAGCTACAGTGAAATCT CGATGGAGTGGGTCCCATCAGTTTGAACAGT TGTCTGGATCCATTTTGTGGATGTAAGAATTG AGGCTATTTTTCCACTGATTAAATTTTTGGCCC TGAGATGCTGCTGAGTTACTAGAAAGTCATTG  AAGGTCTCAACTATAGT-3’ |

**Table S2:** Summary of thermocycling parameters used in this study.

|  | Volume of Solvent (µL) | Initial Hold (min) | Denaturation Temperature (°C) | Denaturation Time (s) | Annealing Temperature (°C) | Annealing Time (s) | qPCR compatible |
| --- | --- | --- | --- | --- | --- | --- | --- |
| Genomic Plant DNA | 0.3 | 10 | 95 | 15 | 65 | 45 | Yes |
| Genomic Plant DNA | 6 | 10 | 95 | 15 | 60 | 45 | Yes |
| Non-target DNA sequence | 0.3 | 2 | 95 | 5 | 60 | 30 | Yes |
| Non-target DNA sequence | 6 | 2 | 95 | 5 | 60 | 30 | Yes |
| Phire Assay | N/A | 5 | 98 | 5 | 62 | 20 | No |

**Table S3:** Summary of all PCR assays used in this study.

| DNA Sequence | MIL or IL | Volume of MIL (µL) | Reaction Volume (µL) | Buffer Type | Primer Concentration (nM) | Additives |
| --- | --- | --- | --- | --- | --- | --- |
| Plant Genomic DNA | N/A | 0 | 20 | SSO Supermix | 200 | None |
| Plant Genomic DNA | [P_6,6,6,14_^+^][Ni(hfacac)_3_^-^], [P_6,6,6,14_^+^][Co(hfacac)_3_^-^], and [P_6,6,6,14_^+^][NTf_2_^-^] | 0.3 | 20 | SSO Supermix | 200 | - 1x Sybr Green |
| Plant Genomic DNA | [P_6,6,6,14_^+^][Ni(hfacac)_3_^-^] | 6 | 20 | SSO Supermix | 200 | - 2x Sybr green - 0.05% Tween20 - 2.5% DMSO - 5 mM MgCl_2_ |
| Plant Genomic DNA | [P_6,6,6,14_^+^][NTf_2_^-^] | 6 | 20 | SSO Supermix | 200 | - 2x Sybr green - 0.05% Tween20 - 2.5% DMSO |
| Plant Genomic DNA | N/A | N/A | 50 | Phire Buffer | 500 | None |
| Non-target DNA | N/A | 0 | 20 | SSO Supermix | 1000 | None |
| Non-target DNA | [P_6,6,6,14_^+^][Ni(hfacac)_3_^-^], [P_6,6,6,14_^+^][Co(hfacac)_3_^-^], and [P_6,6,6,14_^+^][NTf_2_^-^] | 0.3 | 20 | SSO Supermix | 1000 | 1x Sybr Green |
| Non-target DNA | [P_6,6,6,14_^+^][Ni(hfacac)_3_^-^] | 6 | 20 | SSO Supermix | 1000 | - 2x Sybr green - 0.05% Tween20 - 2.5% DMSO - 5 mM MgCl_2_ |
| Non-target DNA | [P_6,6,6,14_^+^][NTf_2_^-^] | 6 | 20 | SSO Supermix | 1000 | - 2x Sybr green - 0.05% Tween20 - 2.5% DMSO |


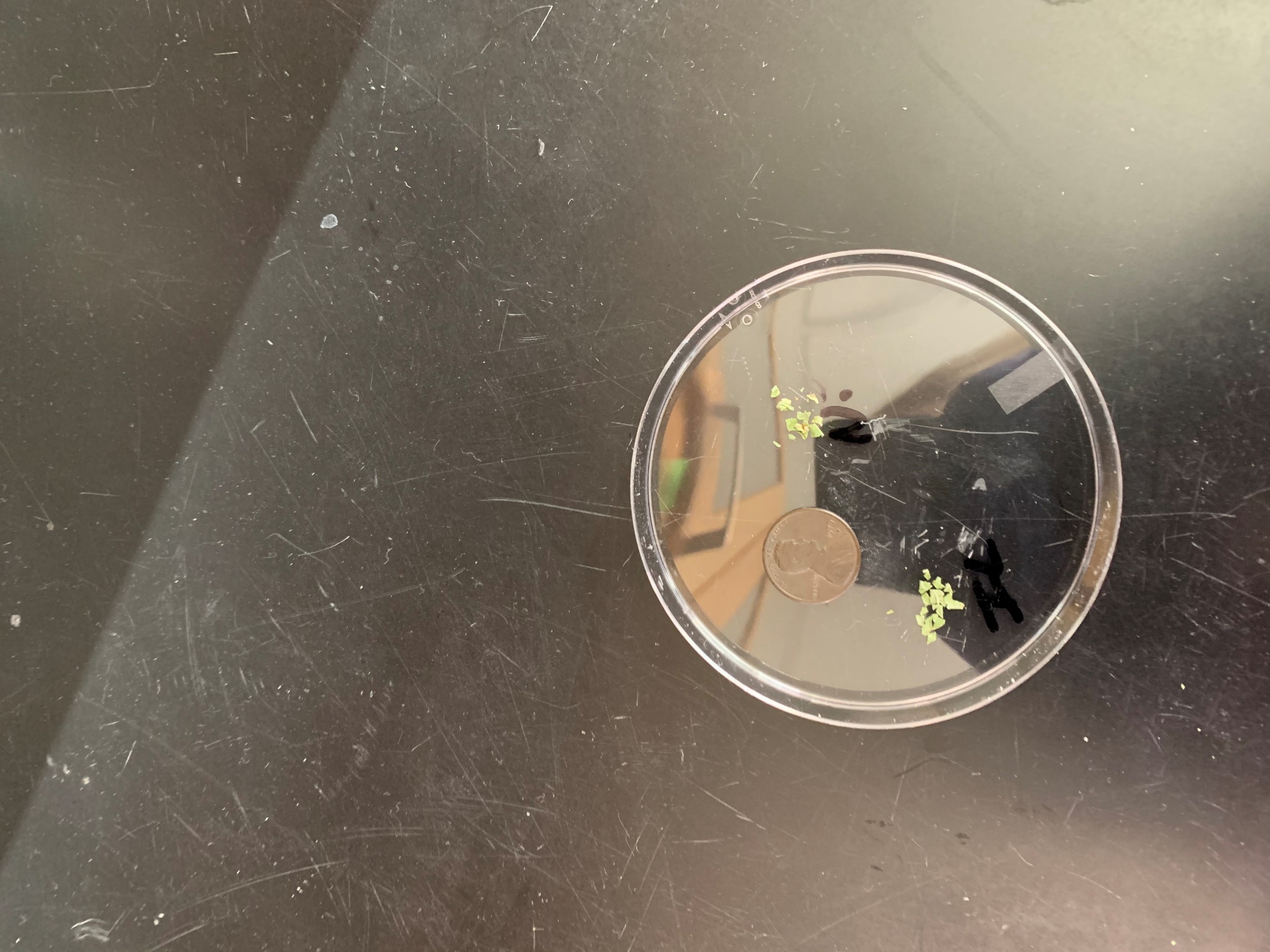


**Figure S1**: Image of the cut-up plant tissue next to a US penny as a scale.

**
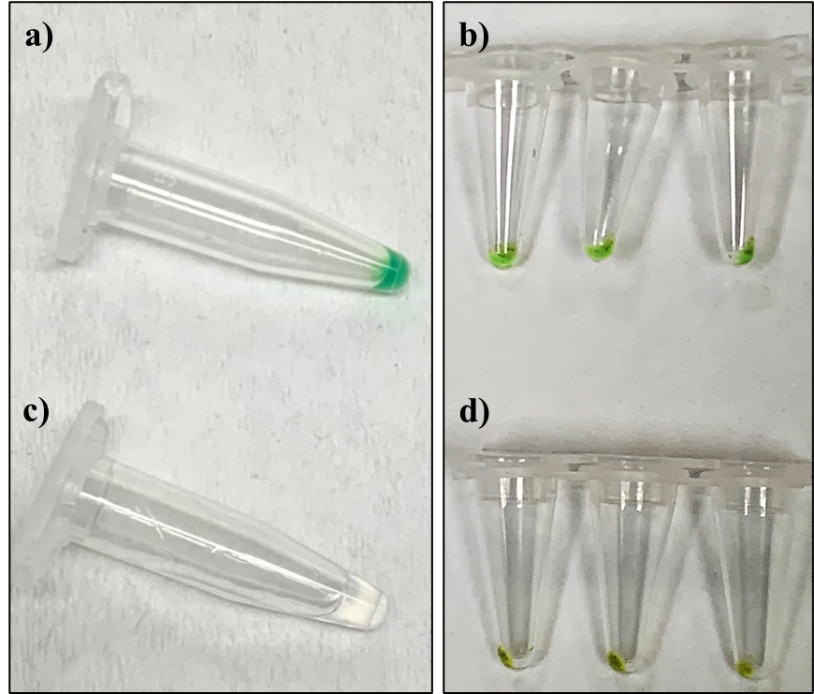
**

**Figure S2**: The a) [P_6,6,6,14_^+^][Ni(hfacac)_3_^-^] MIL and c) [P_6,6,6,14_^+^][NTf_2_^-^] IL prior to cell disruption and extraction, and the b) [P_6,6,6,14_^+^][Ni(hfacac)_3_^-^] MIL and d) [P_6,6,6,14_^+^][NTf_2_^-^] IL after exposing the hydrophobic solvent to 40 mg of ground plant tissue for 30 min.


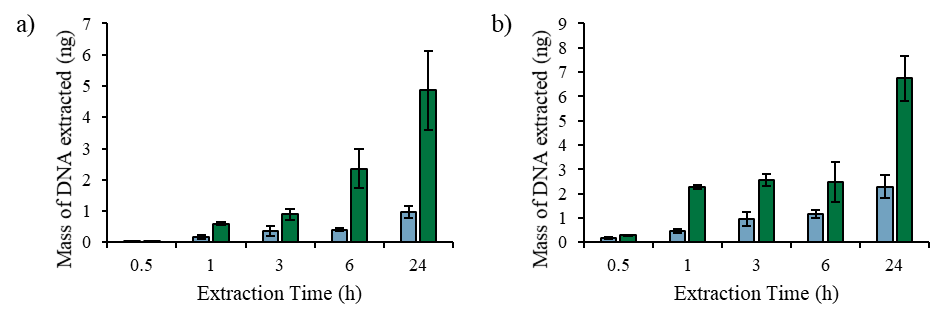


**Figure S3**: DNA extracted from 40 mg of (blue) in-tact and (green) cut-up plant tissue using the (a) [P_6,6,6,14_^+^][Ni(hfacac)_3_^-^] MIL and (b) [P_6,6,6,14_^+^][NTf_2_^-^] IL.


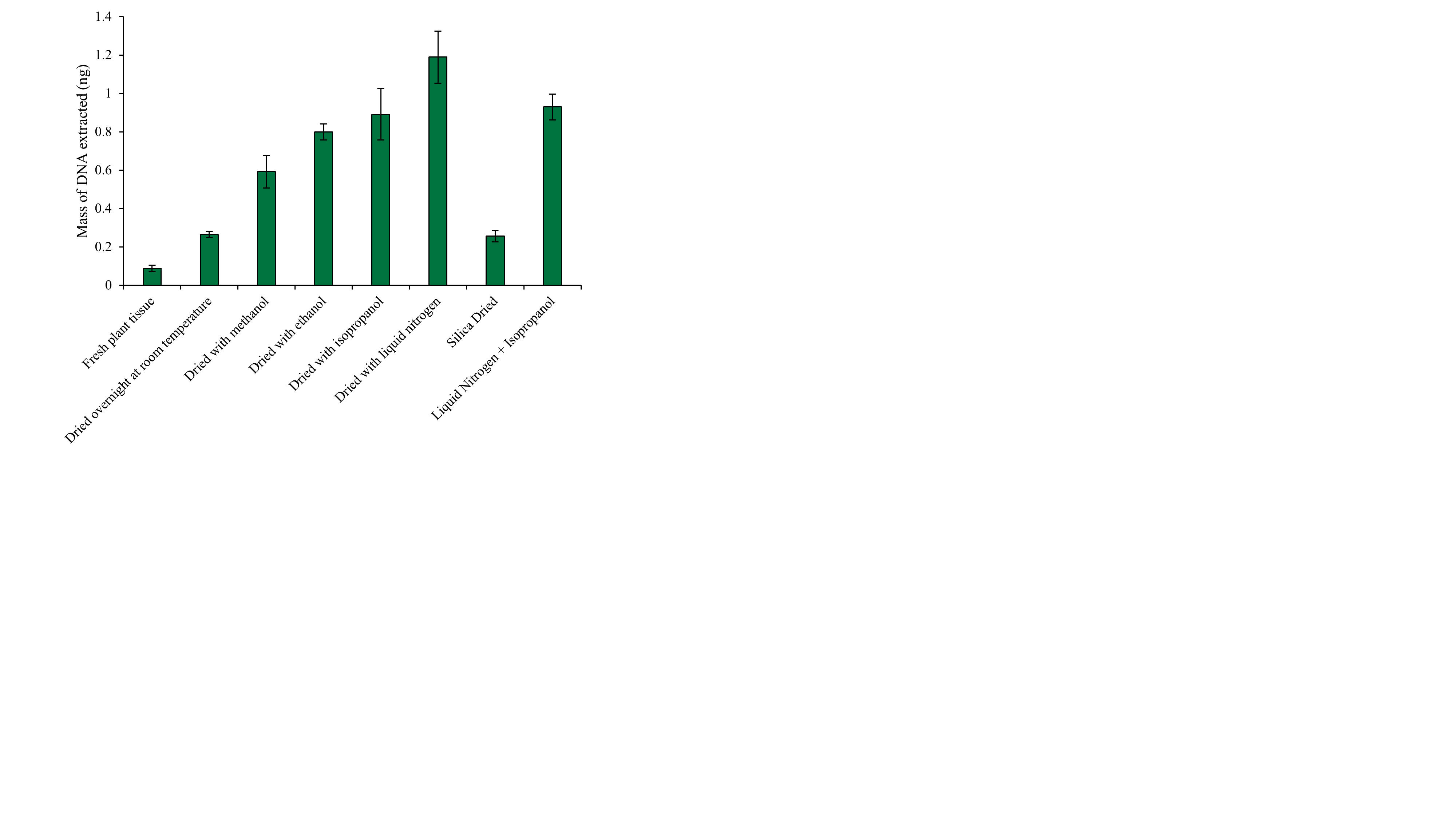


**Figure S4**: Effect of different drying methods on the static lysis method using [P_6,6,6,14_^+^][Ni(hfacac)_3_^-^] MIL. Tissue mass: 40 mg; volume of MIL: 6 µL; incubation time: 1 h.


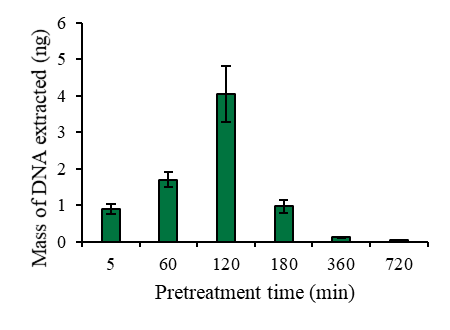


**Figure S5**: Effect of treating plant tissue with isopropanol overtime using the [P_6,6,6,14_^+^][Ni(hfacac)_3_^-^] MIL. Tissue mass: 40 mg; volume of MIL: 6 µL; incubation time: 1 h.


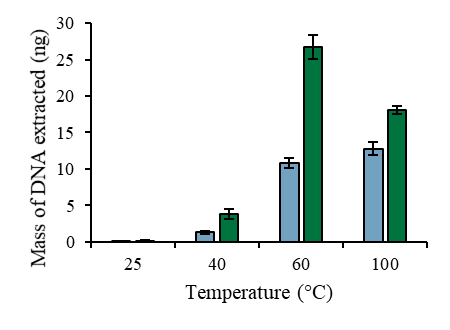


**Figure S6**: Effect of temperature on the lysis of cut-up plant tissue with the (blue) [P_6,6,6,14_^+^][Ni(hfacac)_3_^-^] MIL and (green) [P_6,6,6,14_^+^][NTf_2_^-^] IL. Tissue mass: 40 mg; volume of MIL: 6 µL; incubation time: 0.5 h.


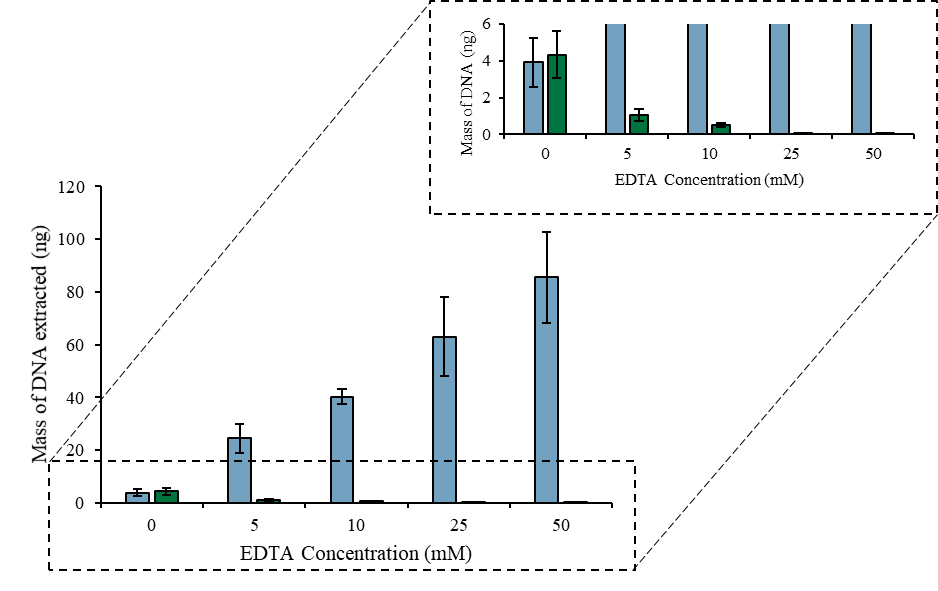


**Figure S7**: EDTA optimization while optimizing the dispersive MIL-lysis and DNA extraction with the (blue) [P_6,6,6,14_^+^][Ni(hfacac)_3_^-^] and (green) [P_6,6,6,14_^+^][Co(hfacac)_3_^-^] MIL. Tissue mass: 40 mg; volume of MIL: 6 µL; vortex time: 1 min; sample volume: 0.5 mL.


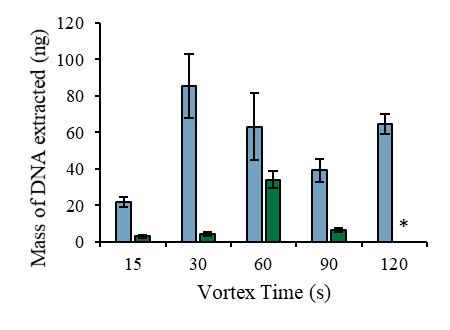


**Figure S8:** Extraction time optimization while optimizing the dispersive MIL-lysis and DNA extraction with the (blue) [P_6,6,6,14_^+^][Ni(hfacac)_3_^-^] and (green) [P_6,6,6,14_^+^][Co(hfacac)_3_^-^] MIL. Tissue mass: 40 mg; volume of MIL: 6 µL; sample volume: 0.5 mL.


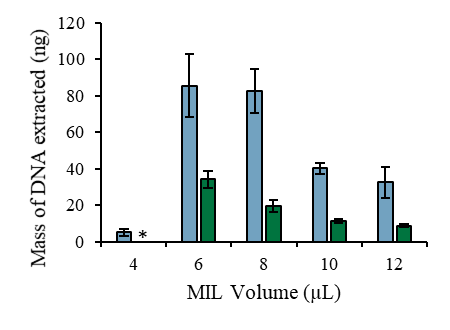


**Figure S9:** Optimization of the volume of MIL dispersed to lyse plant cells and extract genomic DNA using the (blue) [P_6,6,6,14_^+^][Ni(hfacac)_3_^-^] and (green) [P_6,6,6,14_^+^][Co(hfacac)_3_^-^] MIL. Tissue mass: 40 mg; sample volume: 0.5 mL. *Amplification was not observed.


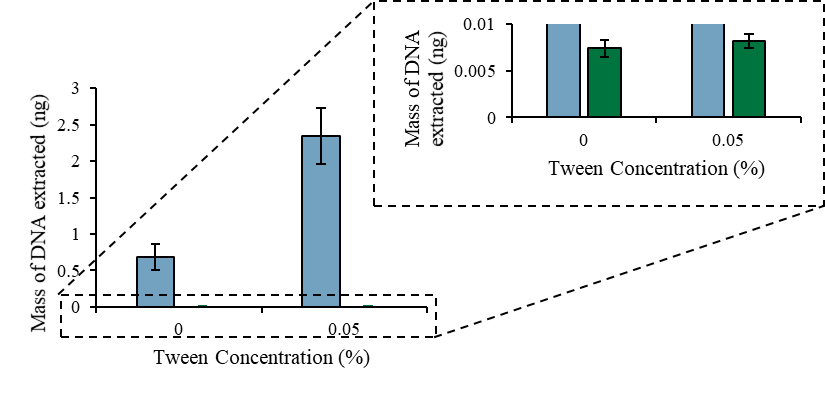


**Figure S10:** Effect of adding Tween20 to the desorption solution aids on the recovery of DNA from the (blue) [P_6,6,6,14_^+^][Ni(hfacac)_3_^-^] and (green) [P_6,6,6,14_^+^][Co(hfacac)_3_^-^] MIL. Tissue mass: 40 mg; sample volume: 0.5 mL; extraction time: 30 s with the [P_6,6,6,14_^+^][Ni(hfacac)_3_^-^] MIL and 60 s with the [P_6,6,6,14_^+^][Co(hfacac)_3_^-^] MIL; desorption time: 10 min.


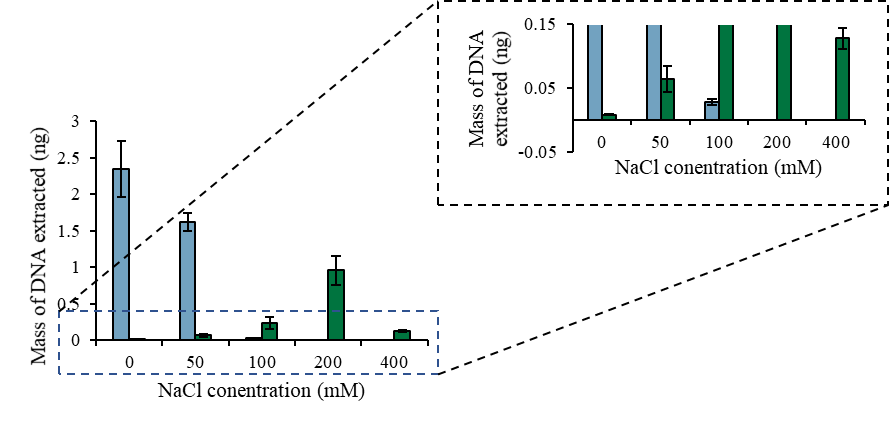


**Figure S1**1: Effect of incorporating sodium chloride to the desorption solution for the recovery of DNA from the (blue) [P_6,6,6,14_^+^][Ni(hfacac)_3_^-^] and (green) [P_6,6,6,14_^+^][Co(hfacac)_3_^-^] MIL. Tissue mass: 40 mg; sample volume: 0.5 mL; extraction time: 30 s with the [P_6,6,6,14_^+^][Ni(hfacac)_3_^-^] MIL and 60 s with the [P_6,6,6,14_^+^][Co(hfacac)_3_^-^] MIL; desorption time: 10 min; desorption solution: 2 mM Tris, 0.05% Tween20.


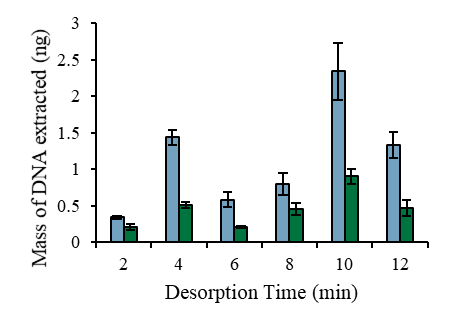


**Figure S12**: Impact of the desorption time towards the recovery of DNA from the (blue) [P_6,6,6,14_^+^][Ni(hfacac)_3_^-^] and (green) [P_6,6,6,14_^+^][Co(hfacac)_3_^-^] MIL. Tissue mass: 40 mg; sample volume: 0.5 mL; extraction time: 30 s with the [P_6,6,6,14_^+^][Ni(hfacac)_3_^-^] MIL and 60 s with the [P_6,6,6,14_^+^][Co(hfacac)_3_^-^] MIL; desorption solution: 2 mM Tris, 0.05% Tween20 with the [P_6,6,6,14_^+^][Ni(hfacac)_3_^-^] MIL and : 2 mM Tris, 0.05% Tween20, 200 mM NaCl with the [P_6,6,6,14_^+^][Co(hfacac)_3_^-^] MIL.


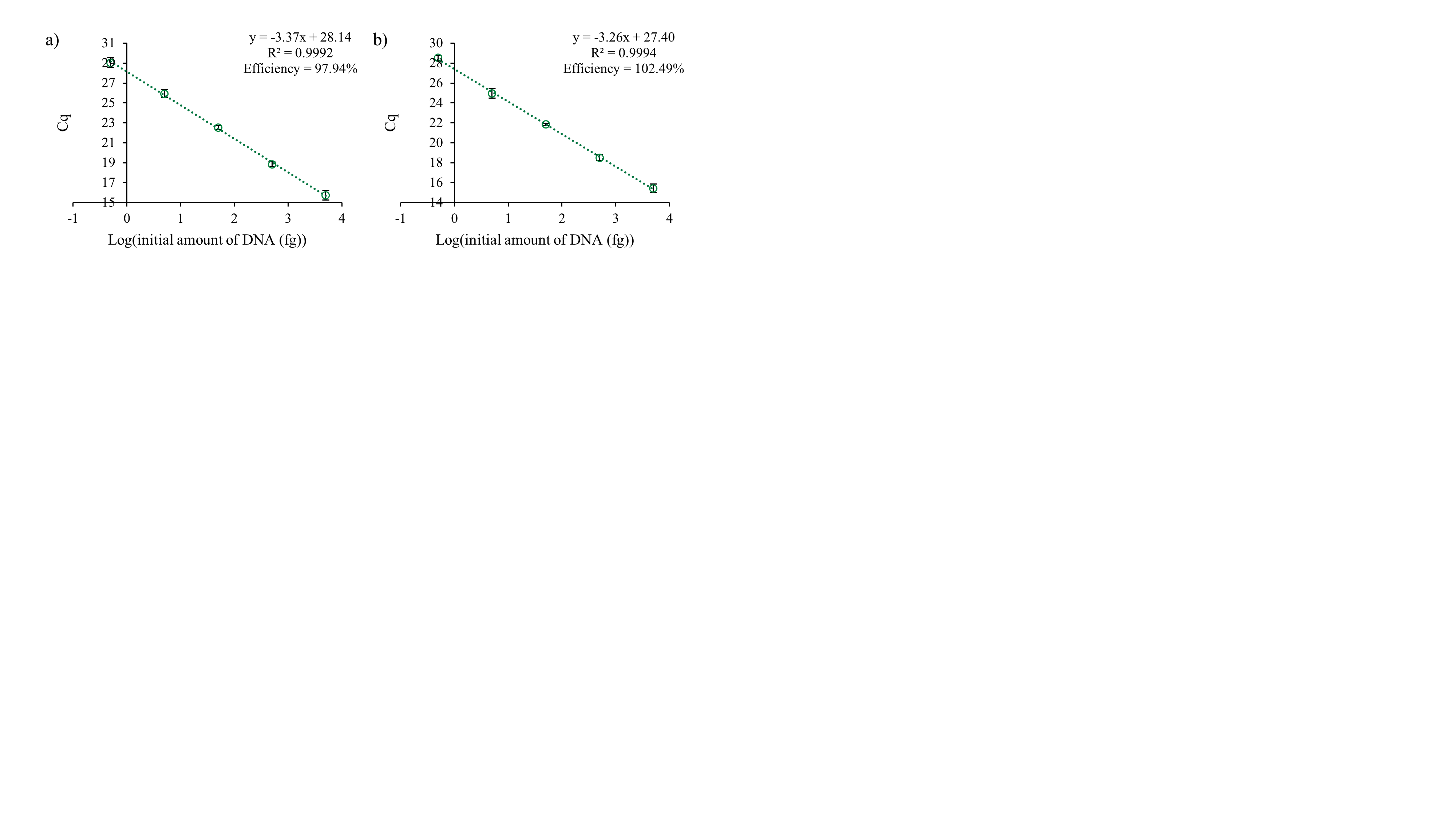


**Figure S13**: Standard curves generated by adding 0.3 µL of the (a) [P_6,6,6,14_^+^][Ni(hfacac)_3_^-^] and (b) [P_6,6,6,14_^+^][Co(hfacac)_3_^-^] MIL that was dispersed in a suspension of 40 mg *A. thaliana* tissue.


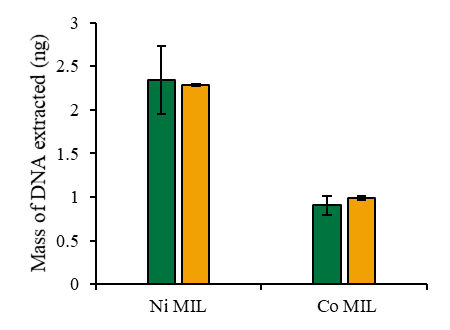


**Figure S14**: Comparison of the amount of DNA recovered from the [P_6,6,6,14_^+^][Ni(hfacac)_3_^-^] and [P_6,6,6,14_^+^][Co(hfacac)_3_^-^] MIL and quantified using (green) qPCR and (orange) Qubit.


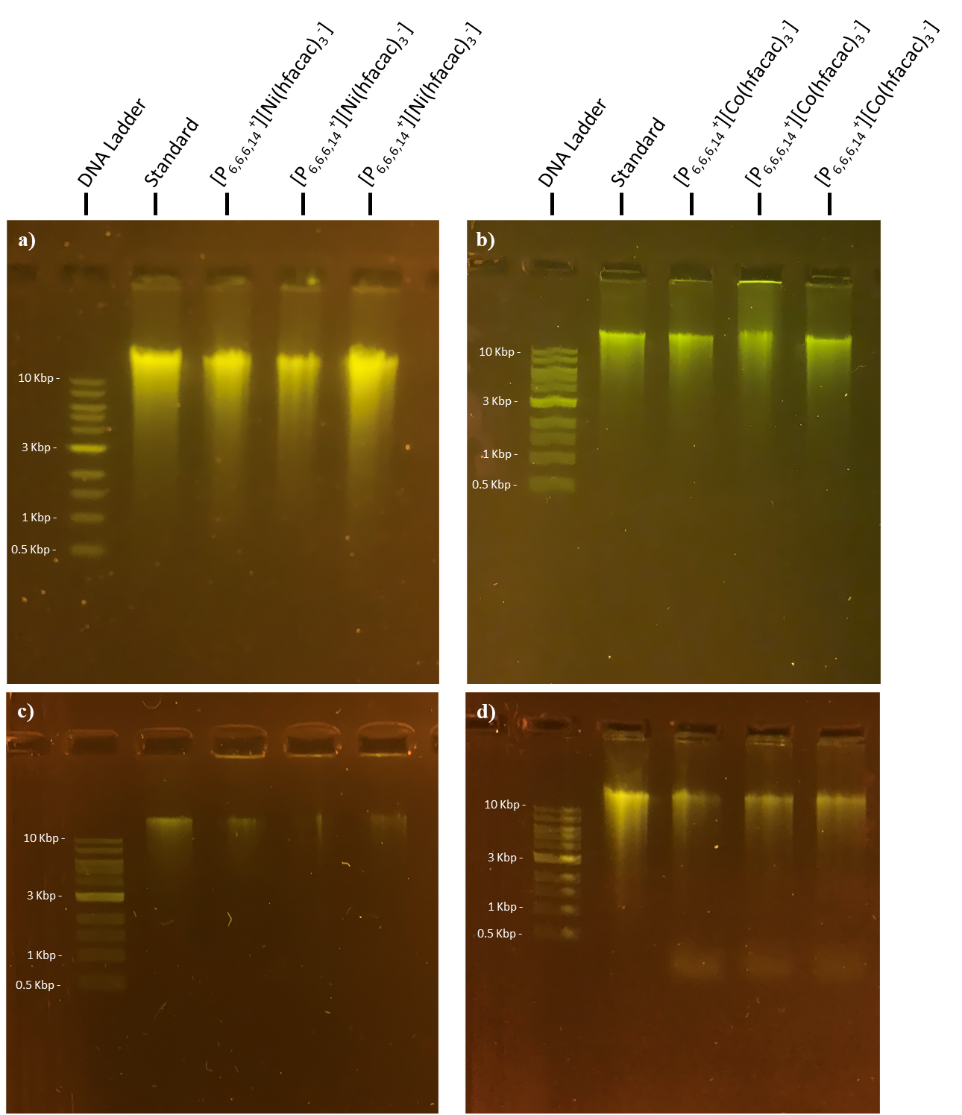


**Figure S15**: Agarose gel separation of and fluorescence detection of stDNA recovered from the (a,c) [P_6,6,6,14_^+^][Ni(hfacac)_3_^-^] and (b,d) [P_6,6,6,14_^+^][Co(hfacac)_3_^-^] MIL after incubating at room temperature for (a,b) 1 h and (c,d) 24 h. DNA was originally isolated from a plant tissue suspension spiked with stDNA.


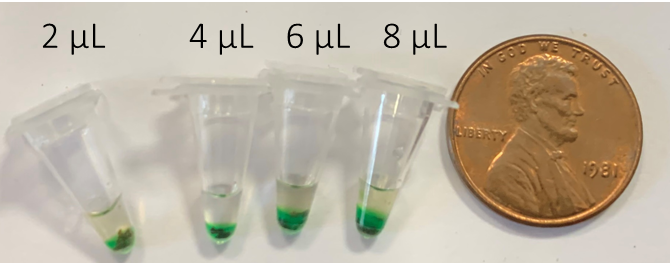


**Figure S16**: Results of adding different volumes of the [P_6,6,6,14_^+^][Ni(hfacac)_3_^-^] MIL and 1 mg of *A. thaliana* tissue to the qPCR assay.


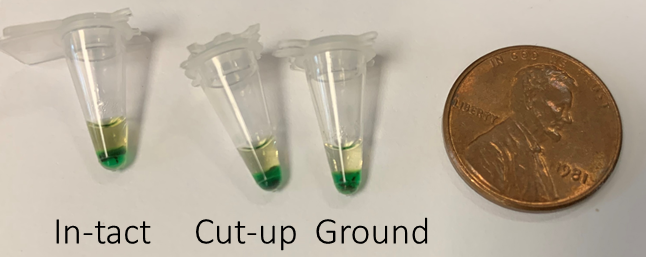


**Figure S17**: The effect of integrating 1 mg of in-tact, cut-up, or ground up *A. thaliana* tissue to the qPCR assay with 6 µL of [P_6,6,6,14_^+^][Ni(hfacac)_3_^-^] MIL.


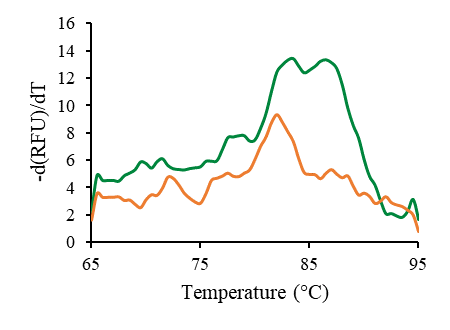


**Figure S18**: Melt curves associated with the (green) successful and (orange) unsuccessful amplification of genomic *A. thaliana* DNA after directly integrating 1 mg of plant tissue and 6 µL [P_6,6,6,14_^+^][Ni(hfacac)_3_^-^] MIL into the qPCR assay.


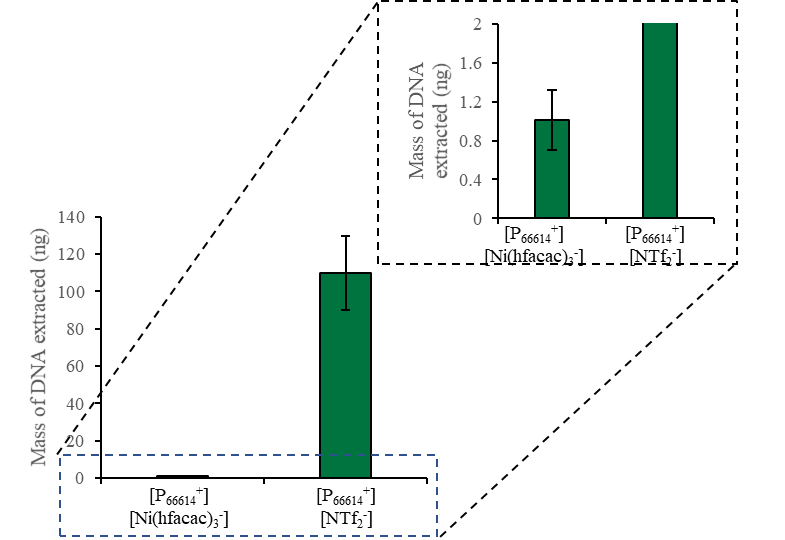


**Figure S19**: Mass of DNA detected when directly integrating 1 mg of fresh *A. thaliana* tissue and 6 µL of MIL or IL into the qPCR assay.


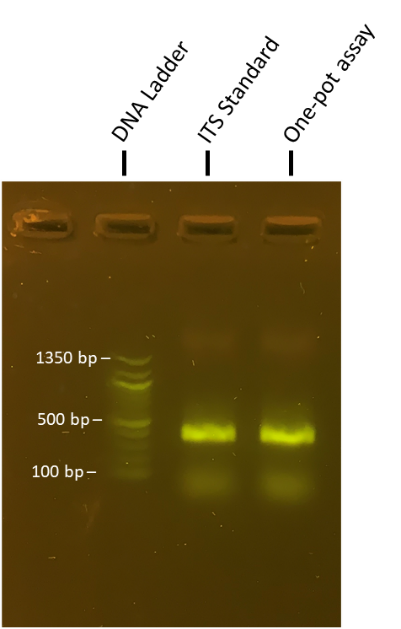


**Figure S20**: Agarose gel detection of the ITS amplicon that was amplified using the Phire assay with 1 mg *A. thaliana* tissue directly integrated into the PCR assay.


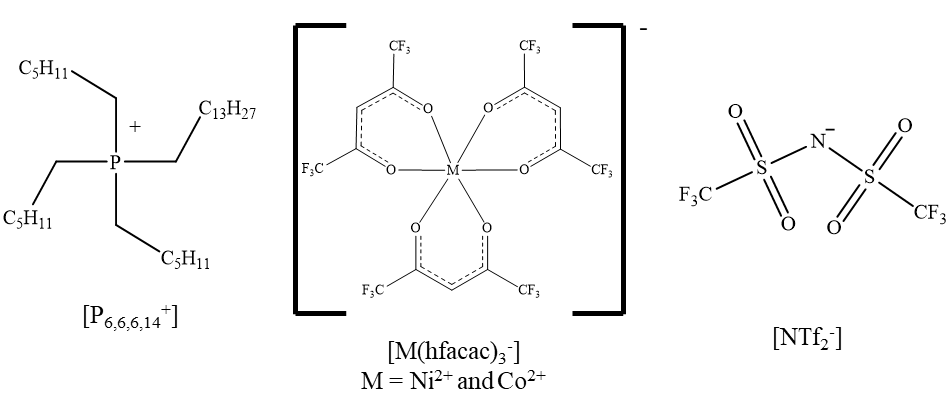


**Figure S21**: Chemical structures of the cations and anions comprising the MILs and IL examined in this study.


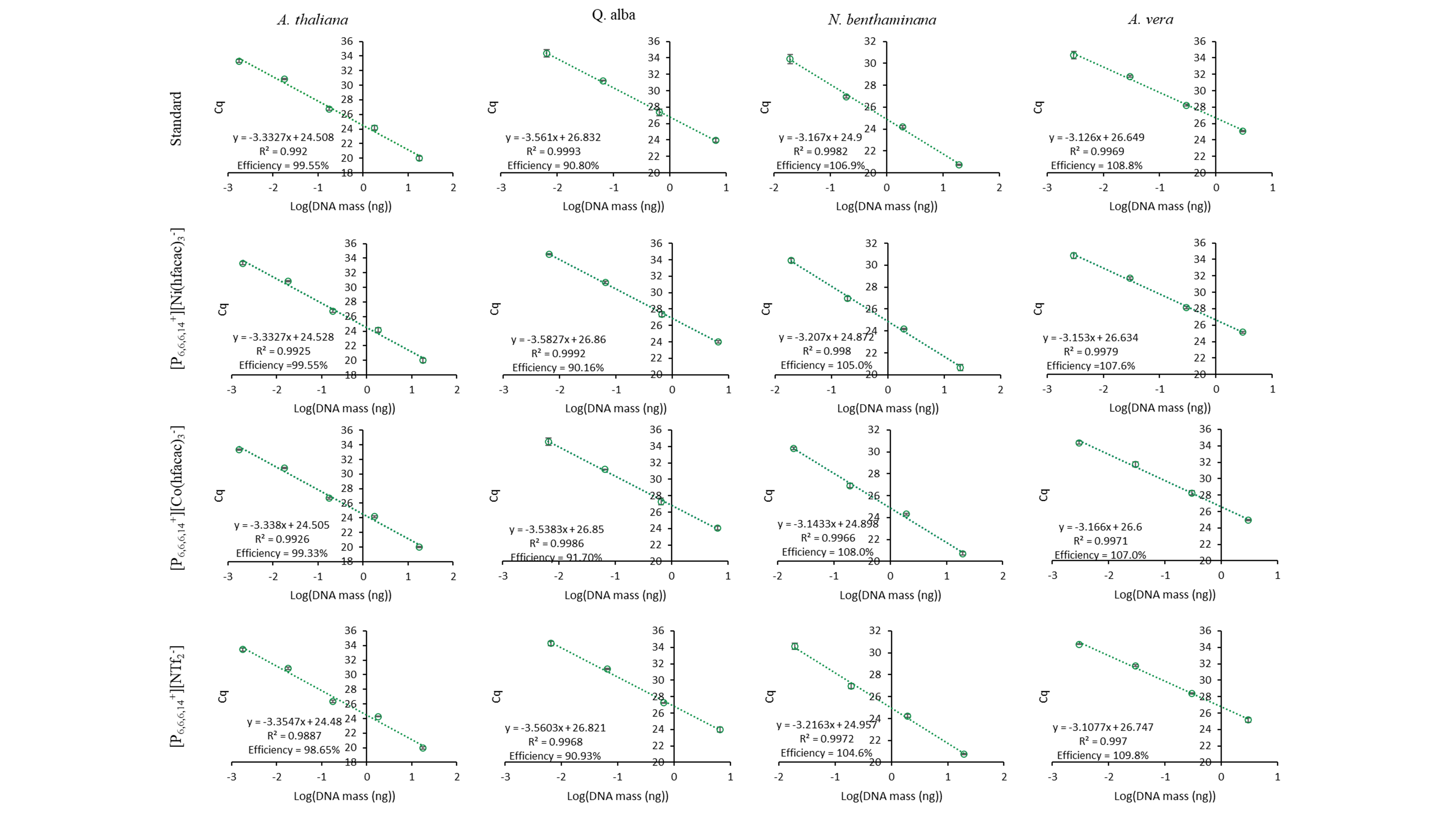


**Figure S22:** Standard curves associated with the qPCR amplification of A. thaliana, white oak, *N. benthaminana,* and *A. vera* with 0.3 µL of [P_6,6,6,14_^+^][Ni(hfacac)_3_^-^] MIL, [P_6,6,6,14_^+^][Co(hfacac)_3_^-^] MIL and [P_6,6,6,14_^+^][NTf_2_^-^] IL.
